# Effector gene silencing coordinated by histone methylation and small RNAs enhances host adaptation in a plant pathogen

**DOI:** 10.1093/nar/gkaf1426

**Published:** 2026-01-08

**Authors:** Liyuan Wang, Xuewei Xiang, Guoyu Yin, Haidong Shu, You Wu, Han Chen, Ren Na, Mark Gijzen, Yingnan Hou, Suomeng Dong

**Affiliations:** Shanghai Collaborative Innovation Center of Agri-Seeds, School of Agriculture and Biology, Shanghai Jiao Tong University, Shanghai 200240, China; State Key Laboratory of Agricultural and Forestry Biosecurity, Key Laboratory of Soybean Disease and Pest Control (Ministry of Agriculture and Rural Affairs), Nanjing Agricultural University, Nanjing 210095, China; Shanghai Collaborative Innovation Center of Agri-Seeds, School of Agriculture and Biology, Shanghai Jiao Tong University, Shanghai 200240, China; State Key Laboratory of Agricultural and Forestry Biosecurity, Key Laboratory of Soybean Disease and Pest Control (Ministry of Agriculture and Rural Affairs), Nanjing Agricultural University, Nanjing 210095, China; State Key Laboratory of Agricultural and Forestry Biosecurity, Key Laboratory of Soybean Disease and Pest Control (Ministry of Agriculture and Rural Affairs), Nanjing Agricultural University, Nanjing 210095, China; Joint International Research Laboratory of Metabolic & Developmental Sciences, School of Life Sciences and Biotechnology, Shanghai Jiao Tong University, Shanghai 200240, China; State Key Laboratory of Agricultural and Forestry Biosecurity, Key Laboratory of Soybean Disease and Pest Control (Ministry of Agriculture and Rural Affairs), Nanjing Agricultural University, Nanjing 210095, China; School of Ecology and Environment, Baotou Teacher’s College, Baotou 014030, China; London Research and Development Centre, Agriculture and Agri-Food Canada, London, Ontario N5V 4T3, Canada; Shanghai Collaborative Innovation Center of Agri-Seeds, School of Agriculture and Biology, Shanghai Jiao Tong University, Shanghai 200240, China; State Key Laboratory of Agricultural and Forestry Biosecurity, Key Laboratory of Soybean Disease and Pest Control (Ministry of Agriculture and Rural Affairs), Nanjing Agricultural University, Nanjing 210095, China

## Abstract

Pathogen adaptability driven by epigenetic processes remains poorly understood and poses a significant challenge to sustainable disease management. Histone 3 lysine 27 trimethylation (H3K27me3) and small RNA (sRNA)-mediated silencing of *avirulence* (*Avr*) genes are two major strategies that pathogens employ to evade recognition by host resistance (R) proteins. Here, we demonstrate that these two epigenetic mechanisms operate in a coordinated manner to silence *Avr* genes in the oomycete *Phytophthora sojae*. CRISPR/Cas9-mediated editing of *PsSu(z)12*, a core component of the Polycomb repressive complex 2 (PRC2), abolished H3K27me3 deposition at *Avr1b* and *Avr3a*, leading to transcriptional reactivation and loss of avirulence. Complementation with *PsSu(z)12* restored H3K27me3 and silencing at *Avr1b*, but not at *Avr3a*. This prompted sRNA profiling at both loci, revealing differential co-enrichment of sRNA and H3K27me3. Integrated analysis of H3K27me3-enriched chromatin immunoprecipitation, RNA and sRNA sequencing data uncovered a strong locus-specific co-silencing pattern, with 11 out of 12 H3K27me3-regulated arginine-X-leucine-arginine effectors also targeted by sRNAs. Notably, epigenetic variation among field isolates indicated regulatory heterogeneity and plasticity in effector control. Together, our findings establish *PsSu(z)12* as a central hub coordinating H3K27me3 and sRNA-mediated effector gene silencing, revealing a dual-layered epigenetic mechanism that enables immune evasion and promotes pathogen adaptation.

## Graphical Abstract

**Graphical Abstract:**
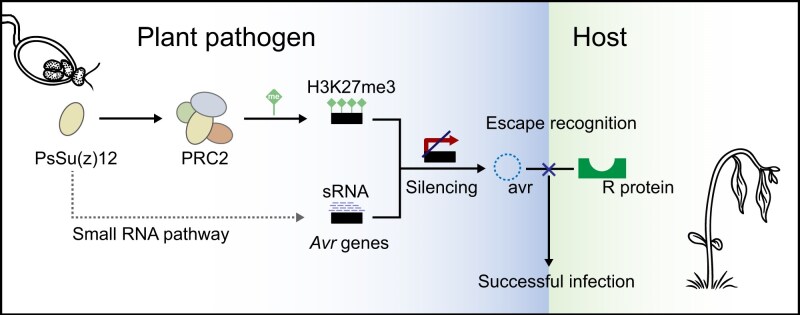

## Introduction

In the mid-20th century, H.H. Flor proposed the gene-for-gene hypothesis [1–3], which posits that for each resistance (*R*) gene in a host plant, there is a corresponding avirulence (*Avr*) gene in the pathogen. Recognition of an Avr effector by its cognate R protein elicits a robust and highly specific immune response, leading to complete resistance. Conversely, loss or inactivation of either component prevents recognition and enables pathogen infection. This model has become a cornerstone of plant immunity research and underpins resistance breeding efforts in crops such as soybean, potato, and wheat [4, 5].

Oomycetes are eukaryotic organisms that resemble filamentous fungi morphologically but are phylogenetically closer to stramenopiles, a group that includes diatoms and brown algae [6, 7]. *Phytophthora sojae*, a representative oomycete species, is the causal agent of soybean stem and root rot, a devastating disease that significantly threatens global soybean production [8–10]. *Phytophthora sojae* secretes a repertoire of arginine-X-leucine-arginine (RxLR) effectors to promote infection, nine of which have been identified as Avr effectors recognized by specific soybean resistance (Rps) proteins, thereby triggering effector-triggered immunity [11].

Widespread and prolonged deployment of single *Rps* genes in agriculture has imposed strong selective pressure on *P. sojae* populations, driving rapid evolution of *Avr* genes through mechanisms such as point mutations, deletions, and gene silencing [4, 9, 11, 12]. These variations allow the pathogen to evade host detection, rendering the corresponding *Rps* genes ineffective. Recent global surveillance has revealed that widely deployed *Rps* genes, such as *Rps1a, Rps1c*, and *Rps1k*, have lost efficacy in major soybean-producing regions [4]. This highlights an urgent need to elucidate the mechanisms underlying *P. sojae Avr* gene variation in order to better understand pathogen adaptation and develop more durable resistance strategies for sustainable management of *Phytophthora* root rot.

Emerging evidence indicates that plant pathogens exploit epigenetic mechanisms, including histone modifications and small RNA (sRNA)-mediated gene silencing, as reversible and dynamic strategies to suppress effector gene expression and evade host immunity [13–17]. For example, transgenerational silencing of the *Avr3a* gene in *P. sojae* is associated with abundant 25 nucleotide (nt) sRNAs across its genomic region, implicating sRNA-directed silencing in the evasion of *Rps3a*-mediated recognition [13]. In contrast, silencing of *Avr1b* in strain P6497 is mediated by histone 3 lysine 27 trimethylation (H3K27me3), thereby enabling evasion of *Rps1b*-dependent immunity [14]. These distinct epigenetic mechanisms underscore the sophisticated strategies employed by *P. sojae* to reprogram its effector repertoire in response to host immune pressures.

RNA interference (RNAi) is a conserved eukaryotic mechanism that employs sRNAs to regulate gene expression. In plants, sRNAs, mainly comprising microRNAs (miRNAs) and small interfering RNAs (siRNAs) of 21–24 nt, act as key regulators of gene expression and genome stability by orchestrating transcriptional gene silencing (TGS) and post-transcriptional gene silencing (PTGS) through diverse and sophisticated biogenesis pathways [18]. Similar RNAi mechanisms exist in animals and fungi, albeit with kingdom-specific adaptations [19, 20]. In contrast, functional characterization of RNAi pathways in oomycetes has lagged, due in part to technical challenges in genetic manipulation. Nevertheless, current evidence indicates that oomycetes possess functional RNAi machinery, including Argonaute (AGO), Dicer-like, and RNA-dependent RNA polymerase proteins, and produce diverse classes of sRNAs that are implicated in transposon silencing, gene regulation, and potentially in host–pathogen interactions [21–25]. In *Phytophthora parasitica*, 25 to 26 nt sRNAs are associated with widespread silencing of endogenous genes and repeat-rich genomic regions, while 21 nt sRNAs predominantly originate from highly expressed genes [26]. A similar pattern has been observed in *Phytophthora infestans*, although the function of 21 nt sRNAs remains unclear [26]. In *P. sojae*, 24–26 nt sRNAs present the dominant class and show strong association with *Avr* gene silencing [13, 27]. These findings underscore the evolutionary conservation and functional diversification of sRNA-mediated silencing pathways in oomycetes and highlight their potential roles in genome regulation and pathogenicity.

H3K27me3 is a well-characterized epigenetic mark catalyzed by the Polycomb repressive complex 2 (PRC2), which compacts chromatin, inhibits transcription, and maintains gene silencing [28]. In *P. sojae*, PRC2 comprises four conserved subunits: E(Z), Su(z)12, ESC, and Nurf55 [14]. Notably, studies in *Drosophila* have revealed mechanistic crosstalk between sRNA-directed PTGS and Polycomb-mediated TGS, wherein RNAi components including Dicer-2, PIWI, and AGO1 contribute to Polycomb-mediated silencing [29, 30]. More recent studies have demonstrated that PRC2 can bind nascent RNA, which facilitates its recruitment to target loci and stabilizes its association with chromatin, thereby enhancing transcriptional repression [31–33], and that Su(z)12 can bind RNA independently of other PRC2 subunits [34, 35]. Notably, RNA degradation has been shown to enhance the interaction of PRC2 with chromatin [34]. In *Tetrahymena thermophila*, the EZL1 complex, a Polycomb repressive complex containing PRC1- and PRC2-like components, is recruited in a nuclear RNAi-dependent manner and is required for H3K27/H3K9 methylation, heterochromatin formation, siRNA production, and genome rearrangement, thereby functionally linking Polycomb repression with RNA silencing pathways [36]. These findings suggest that RNA-based pathways may coordinate with histone modification machinery to establish flexible and reversible regulatory programs during pathogen–host interactions.

In *P. sojae* strain P6497, where *Avr1b* is transcriptionally silenced by H3K27me3, a detectable level of *Avr1b*-associated sRNAs also accumulated [27], suggesting potential cooperation between histone modification and sRNA-mediated silencing. In contrast, the *Avr3a*-silenced strain ACR10 exhibits a markedly higher abundance of *Avr3a*-targeting sRNAs compared to strains such as P6497 [13, 37, 38]. However, whether histone modifications also contribute to the *Avr3a* silencing in coordination with sRNAs has remained unresolved. In this study, we demonstrate that H3K27me3 and sRNAs act in a coordinated manner to silence *Avr1b, Avr3a*, and additional RxLR effector genes in *P. sojae*. Genetic disruption of *PsSu(z)12*, a core subunit of PRC2, led to the loss of H3K27me3, transcriptional reactivation, and concurrent depletion of sRNA accumulation at effector loci. Notably, the epigenetic regulation of the RxLR effector repertoire by H3K27me3 and sRNAs differs among field isolates, reflecting regulatory heterogeneity and adaptive plasticity. These findings uncover a previously unrecognized coordination between histone methylation and sRNA pathways in *P. sojae*, and establish a dual-layer epigenetic mechanism that underlies immune evasion and promotes host adaptation in *P. sojae*.

## Materials and methods

### *Phytophthora sojae* strains and culture

*Phytophthora sojae* strains P6497 and sc10 were sourced from the *Phytophthora* culture collection at Nanjing Agricultural University, while strain ACR10 was obtained from Agriculture and Agri-Food Canada, London, ON. The *P. sojae* strains were routinely cultured at 25°C in the dark on 10% vegetable (V8) juice agar medium. For non-sporulating hyphae, cultures were grown in 10% V8 liquid medium at 25°C in the dark, and hyphal tissues were collected after three days of cultivation.

### Plant cultivation and virulence assay

Soybean [*Glycine max* (L.) Merr.] cultivars *Williams, L77-1863* (*Rps1b*), and *L83-570* (*Rps3a*) were obtained from a collection at Agriculture and Agri-Food Canada (Harrow, Ontario). *Williams* is a susceptible cultivar that lacks major *Rps* resistance genes and was used as a control. *L77-1863 and L83-570* are near-isogenic lines in the *Williams* background, carrying the *Rps1b* and *Rps3a* resistance genes, respectively. These cultivars served as standard differential hosts to assess the specificity of interactions between *P. sojae* strains and soybean lines carrying different *Rps* genes. Virulence assays were conducted on 7-day-old soybean seedlings grown under light conditions using the hypocotyl split inoculation method [39]. Each 10 cm diameter plastic pot contained ~10 plants. *P. sojae* strains were pre-cultured on 10% (*v/v*) V8 agar plates for 3–5 days. Agar plugs (2 × 4 mm) were cut from the advancing edge of the mycelial colonies and inoculated into the splits of the hypocotyls. After inoculation, the seedlings were kept in a greenhouse with high humidity for 12 h. Disease symptoms were documented three days post-inoculation by photographing the plants. Each *P. sojae* strain was evaluated in three independent biological replicates.

### RNA extraction and mRNA quantification

Total RNA was extracted using the TRIzol Reagent (Invitrogen) following the manufacturer’s protocol. RNA quality and concentration were assessed by 1% agarose gel electrophoresis and a Nanodrop ND-1000 spectrophotometer. First-strand complementary DNA (cDNA) was synthesized using the PrimeScript RT reagent Kit with gDNA eraser (Takara). Gene expression was analyzed using either standard PCR, to determine the on/off transcriptional status of effector genes, or quantitative PCR (qPCR), to quantify relative transcript levels. qPCR reactions were performed on a *qTOWER3* real-time PCR system (Analytik Jena, Germany). with the following conditions: 95°C for 30 s, followed by 40 cycles of 95°C for 5 s and 60°C for 34 s. Dissociation curves were generated to verify specificity. *Actin* was used as the endogenous reference, and relative transcript levels were calculated using the 2^−ΔΔCt^ method. The primers used are listed in Supplementary Table S1.

### *Phytophthora sojae* gene editing and complementation

CRISPR/Cas9-mediated editing of *PsSu(z)12* in *P. sojae* strain ACR10 was performed via polyethylene glycol (PEG)-mediated protoplast transformation [14, 40, 41]. Two single guide RNAs (sgRNAs) targeting unique coding regions were designed: sgRNA207 (5′-GCTGCCACGCCGGGTCCAGG-3′) and sgRNA768 (5′-GCAGAGGATCCCAAGTAAGA-3′). Each sgRNA was flanked by hammerhead ribozyme sequences and cloned into the pYF2.3G-Ribo-sgRNA vector via NheI/BsaI sites.

For transformation, 35 µg of plasmid DNA was incubated with 2 × 10^6^ freshly prepared protoplasts in 1.74 ml of PEG–CaCl₂ solution and incubated at 4°C for 20 min to facilitate uptake. Treated protoplasts were regenerated overnight in liquid pea/0.5 M mannitol (PM) medium, then mixed with molten PM agar containing 50 µg/ml G418 and plated. After two days at 25°C, a layer of 10% V8 agar with G418 was overlaid to enhance selection. Emerging transformants were transferred to 10% V8 agar supplemented with G418 and screened by PCR, followed by Sanger sequencing. Primer sequences are listed in Supplementary Table S1.

For complementation, the *PsSu(z)12-*edited mutant A13 was used as the recipient strain. *In situ* complementation was performed by homology-directed repair (HDR). A donor plasmid was constructed in pBluescript SK II (+) containing 1-kb homologous arms flanking the A13 deletion region and carrying a synonymous C768T substitution within the target site to prevent repeated cleavage by Cas9, which also served as a diagnostic marker for complementation. The HDR donor plasmid was co-transformed with a Cas9 expression vector carrying an sgRNA (A13_sgRNA207: 5′-GCAGAGGATCCCAAGTAAGG-3′) targeting the deletion site, using PEG-mediated protoplast transformation. Transformants were selected on 10% V8 agar supplemented with G418 (50 µg/ml), as the parental mutant A13 had lost G418 resistance. Complemented strains were verified by PCR amplification across the edited locus and confirmed by Sanger sequencing, which detected precise integration of the donor sequence with the synonymous substitution. Primer sequences are listed in Supplementary Table S1.

### Synthetic dsRNA transfection assay in *P. sojae*

To assess the functional effect of sRNAs on *Avr3a* silencing, synthetic double-stranded RNAs (dsRNAs) corresponding to a highly abundant 25 nt or overlapping 21 nt sRNA from the *Avr3a* locus were introduced into the *PsSu(z)12* mutant strain A13 via lipid-mediated transfection. Nuclease-free water (NF-water) and non-targeting control (NC) dsRNA treatments served as negative controls. Oligo sequences are listed in Supplementary Table S1.

Mycelia of strain A13 were cultured in liquid Nutrient Pea Broth (NPB) medium for three days at 25°C in darkness before collecting. Protoplasts were prepared by enzymatic digestion and resuspended in MT buffer (1 M mannitol, 10 nM Tris/HCl, pH 7.5, 20 mM CaCl_2_) to a final concentration of 1 × 10^7^ protoplasts/ml. For each transfection, 1.5 μl of Lipofectamine^®^ RNAiMAX Reagent (Invitrogen) was diluted in 25 μl MT buffer, and 0.5 μl (5 pmol) of dsRNA (10 μM stock solution) was diluted in a separate 25 μl of MT buffer. The two solutions were mixed thoroughly and incubated at room temperature for 5 min. Then, 50 μl of the lipoplex mixture was added to the prepared 50 μl protoplast suspension and incubated at 25°C in darkness for 24 h. Subsequently, 2 ml of PM medium was added to each well and cultured for an additional four days. Finally, mycelia that regrew from transfected protoplasts were harvested for RNA extraction. *Avr3a* expression levels were quantified by RT-qPCR. Transcript abundance was normalized to the *Actin* reference gene using the 2^−ΔΔCt^ method.

### RNA sequencing and data analysis

RNA sequencing (RNA-seq) was performed by BGI (Shenzhen, China) and Genome Quebec (Quebec, Canada), and clean reads were delivered. We further filtered the reads using Trimmomatic [42] and aligned the clean reads to the *P. sojae* P6497 genome assembly v3.0 (Ps3.0) using Hisat2 [43] and converted using SAMtools [44]. The resulting binary alignment map (BAM) files were normalized using bins per million mapped reads (BPM) with deepTools [45], and biological replicates were merged and visualized using Integrative Genomics Viewer (IGV) [46]. The transcripts per million (TPM) value was calculated using StringTie [47]. DESeq2 [48] was subsequently employed to identify differentially expressed genes (DEGs), with the adjusted *P*-value threshold set to .05 and the log_2_ fold change threshold (lfcThreshold) parameter was used to define the corresponding fold-change significance. RNA-seq data for sc10, ACR10, A13, and H7, each with two biological replicates, were submitted to NCBI under BioProject PRJNA1206799. The RNA-seq data for P6497 and T34 were accessed from NCBI under accession numbers GSE116089 and GSE127207.

### Chromatin immunoprecipitation sequencing and data analysis

Chromatin immunoprecipitation (ChIP) experiments were performed using a micrococcal nuclease (MNase)-based protocol adapted for *P. sojae*. For nuclei preparation, 1.5 g of 3-day-old mycelia were harvested and ground in liquid nitrogen. The frozen powder was resuspended in 20 ml of buffer M1 buffer (0.1 M potassium phosphate, pH 7.0, 0.1 M NaCl, 0.0781% β-mercaptoethanol, and 11.85% hexylene glycol) and incubated for 10 min. The homogenate was filtered through double-layered Miracloth, and nuclei were pelleted by centrifugation and washed twice with 5 ml of M2 buffer (0.1 M potassium phosphate, pH 7.0, 0.1 M NaCl, 10 mM MgCl₂, 0.078% β-mercaptoethanol, 11.8% hexylene glycol, 0.5% Triton X-100), followed by a final wash with 5 ml of MNB buffer (10% sucrose, 50 mM Tris–HCl, pH 7.5, 4 mM MgCl₂, and 1 mM CaCl₂). The resulting pellet was gently resuspended in 1 ml of MNB buffer. Chromatin digestion was carried out using MNase (NEB, M0247S) at 37°C for 10 min. The reaction was terminated by adding 10% volume of 0.5 M EDTA (ethylenediaminetetraacetic acid). Digestion efficiency was assessed by agarose gel electrophoresis, confirming predominant enrichment of dinucleosomal DNA. After centrifugation, 40 μl of supernatant was collected as input and processed by phenol extraction. The remaining chromatin was incubated with anti-H3K27me3 antibodies (Millipore, 07–449) for 6 h at 4°C, followed by binding to pre-washed protein A Dynabeads (Thermo Fisher, 10001D) for an additional 6 h. Immunoprecipitates were sequentially washed with buffer A (50 mM Tris–HCl, pH 7.5, 10 mM EDTA) containing 50, 100, or 150 NaCl. Bound chromatin was eluted with 400 μl of elution buffer (20 mM Tris–HCl, pH 7.5, 50 mM NaCl, 5 mM EDTA, 1% sodium dodecyl sulfate) at 65°C for 15 min. DNA was purified by phenol-chloroform extraction and ethanol precipitation and resuspended in NF-water for downstream applications.

For ChIP-seq, sequencing libraries were prepared and subjected to high-throughput sequencing by BGI (Shenzhen, China) and Genome Quebec (Quebec, Canada). Initial raw reads were filtered using FASTX-Toolkit (https://github.com/agordon/fastx_toolkit) and Trimmomatic. Subsequently, the filtered reads were aligned to the *P. sojae* v3.0 using Bowtie2 [49] and converted to BAM files via SAMtools. Broad peak calling was executed using MACS3 [50] with parameters set to --broad-cutoff 0.05, --extsize 147, and --keep-dup all. The MACS3 bdgcmp module was employed to calculate IP/Input comparison scores using the fold enrichment (FE) function, combining biological replicates to generate a normalized measure of signal intensity. The resulting bdg files were transformed into bw files and visualized using IGV. Differential peaks were identified using DiffBind [51]. Read counts were quantified using the dba.count module with the parameters summits=TRUE, score=DBA_SCORE_TMM_READS_FULL, filter=FALSE, and bRemoveDuplicates=FALSE. Results were obtained with an adjusted *P*-value of .05 and an lfcThreshold defining fold-change significance. Peaks were annotated using ChIPseeker [52], and associated genes were supplemented using the Bedtools [53] intersect function to account for cases where a broad peak intersects with multiple genes. Because H3K27me3 forms broad domains that can span across adjacent genes, the number of annotated genes may exceed the number of peaks.

The ChIP-seq data for P6497 and T34 were accessed from NCBI under accession number GSE127206. For sc10, ACR10, A13, and H7, H3K27me3 immunoprecipitation samples were prepared in triplicate, with input samples comprising one replicate for ACR10 and two replicates for the other strains. The data were submitted to NCBI under BioProject PRJNA1206799.

### Small RNA sequencing and data analysis

Small RNA sequencing (sRNA-seq) was performed by BGI (Shenzhen, China), and clean reads were delivered. The sequencing reads were filtered using Cutadapt [54], with 21–28 nt sRNAs retained for subsequent analyses. Filtered reads were then aligned to *P. sojae* v3.0 using Bowtie [55], allowing up to one mismatch (-v 1). The resulting SAM files were converted to BAM files using SAMtools, normalized using DeepTools bamCoverage with the BPM method, merged across biological replicates, and visualized using IGV. Quantification of read counts at loci of interest was conducted using ShortStack [56] and calculated in reads per million mapped (RPMM). Differential expression analysis of sRNAs was performed using DESeq2, applying an adjusted *P*-value threshold of <.05 and an lfcThreshold to determine significance.

Three biological replicates were conducted for sc10 and A13, while two replicates were performed for P6497, T34, and H7. The data was submitted to NCBI under BioProject PRJNA1206799. As for ACR10, three biological replicates of ACR10 were downloaded from NCBI Bioproject PRJNA300858.

## Results

### H3K27me3-mediated silencing of Avr effectors determines virulence specificity

**Figure 1.**
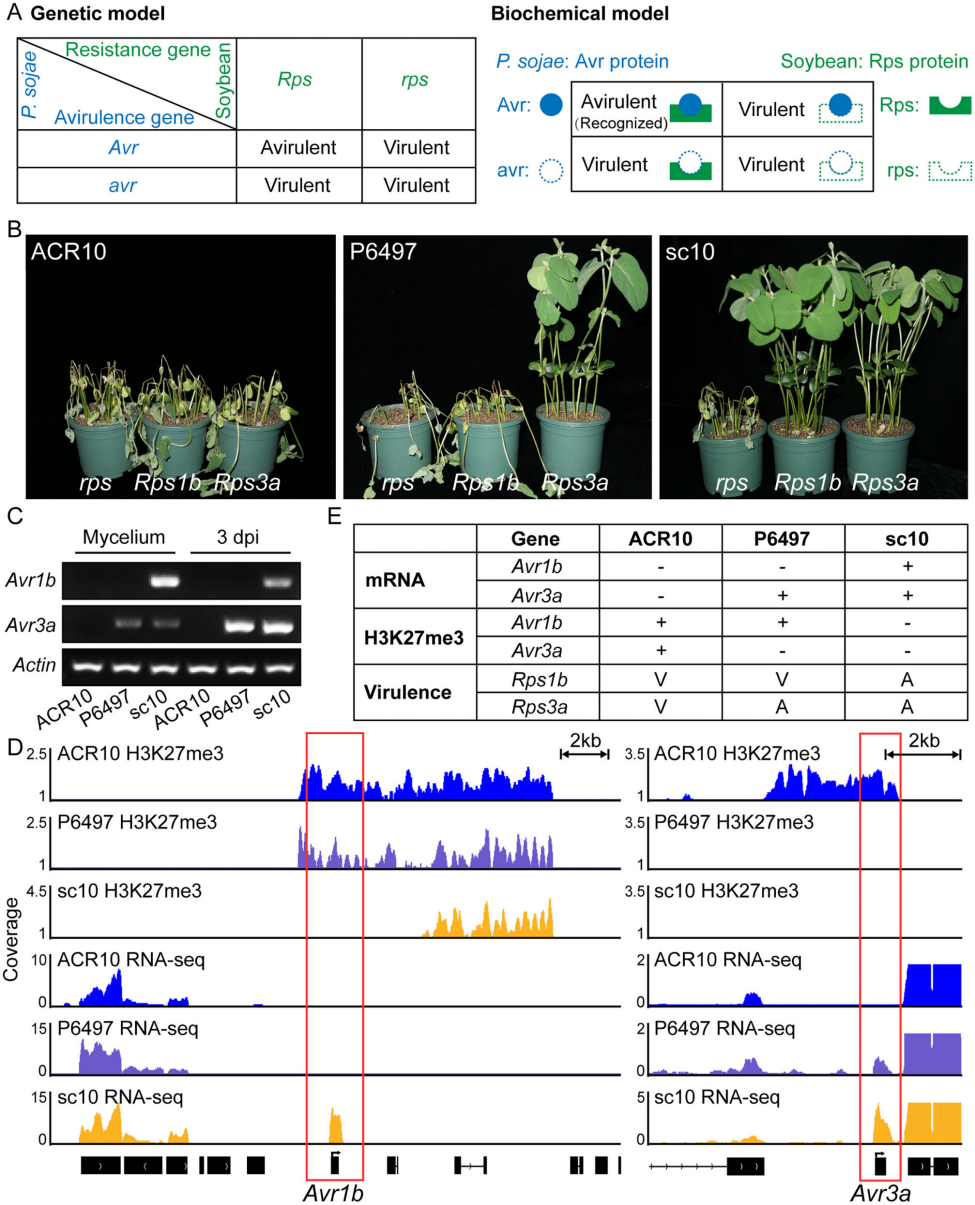
H3K27me3 modification at the *Avr1b* and *Avr3a* loci underpins the adaptation of *P. sojae* strains to specific soybean cultivars. (**A**) Conceptual model of the gene-for-gene interaction between *P. sojae* and soybean, illustrating how *Avr*-*R* gene recognition determines infection outcomes from the pathogen’s perspective. (**B**) Virulence assays of *P. sojae* strains ACR10, P6497, and sc10 on soybean cultivars *Williams* (*rps*), *L77-1863* (*Rps1b*), and *L83-570* (*Rps3a*). Photographs were taken three days post-hypocotyl inoculation. Results were consistent across three biological replicates. (**C**) RT-PCR analysis showing the *Avr1b* and *Avr3a* expression in mycelia and infected tissues (three days post-inoculation). *Actin* served as a positive control. Three biological replicates were conducted, yielding similar results. (**D**) H3K27me3 ChIP-seq and RNA-seq profiles at the *Avr1b* and *Avr3a* loci in the three strains are visualized in the IGV browser. H3K27me3 coverage was normalized to input as FE using MACS3 with three biological replicates. The coverage of RNA-seq reads was normalized as BPM and presented as mean values of two biological replicates. (**E**) Summary of *Avr1b*/*Avr3a* expression, H3K27me3 enrichment, and virulence phenotypes in ACR10, P6497, and sc10. “−” indicates absence of transcript or mark; “+” indicates presence. V and A indicate virulent or avirulent outcomes on soybean cultivars carrying the *Rps1b* or *Rps3a*, respectively.

In gene-for-gene interactions between *P. sojae* and soybean, *Avr* effector genes in the pathogen can be recognized by cognate *Rps* proteins in the host, triggering immunity and preventing infection. When *Avr* genes are absent or silenced, recognition fails and the strain becomes virulent. This relationship is illustrated in Fig. 1A, which summarizes both the classical genetic model and the underlying biochemical basis of effector–receptor recognition.

To characterize strain-specific virulence, we examined three naturally occurring *P. sojae* strains, ACR10, P6497, and sc10, on soybean cultivars carrying different *Rps* genes. ACR10 displayed the broadest virulence, causing disease on both soybean cultivars harboring *Rps1b* (*L77-1863*) and *Rps3a* (*L83-570*). P6497 was virulent on *Rps1b* plants but not on those carrying *Rps3a*, whereas sc10 was avirulent on both cultivars. Meanwhile, all three strains successfully infected *Williams*, which lacks *Rps1b* and *Rps3a* (Fig. 1B). These contrasting phenotypes suggest genetic or epigenetic variation at the *Avr1b* and *Avr3a* loci underlie the strain-specific interactions with soybeans.

Polymerase chain reaction (PCR) followed by Sanger sequencing confirmed that the coding sequences of *Avr1b* and *Avr3a* are, respectively, identical across the three strains (Supplementary Fig. S1), thereby excluding sequence polymorphisms as the basis for the observed phenotypic differences. Transcriptome analysis of the three strains revealed that only *Avr1b* and *Avr3a* among all known *Avr* effector genes showed strain-specific transcriptional variation (Supplementary Fig. S2). To validate these observations at the transcriptional level, we performed reverse transcription PCR (RT-PCR) using RNA extracted from mycelia and infected soybean seedlings at three days post-inoculation. The results showed that in ACR10, both *Avr1b* and *Avr3a* were transcriptionally silenced at both stages, whereas in sc10, both genes were actively expressed. In contrast, P6497 exhibited silencing of *Avr1b* but maintained consistent expression of *Avr3a* (Fig. 1C). These transcriptional patterns aligned with the strain-specific virulence profiles, suggesting that the observed differences are likely governed by regulatory variation, possibly involving epigenetic mechanisms, rather than by coding-sequence polymorphisms.

Notably, our previous work demonstrated that silencing of *Avr1b* in P6497 is maintained by H3K27me3 deposition [14], prompting us to examine whether this epigenetic mark also underlies the simultaneous silencing of *Avr1b* and *Avr3a* in ACR10. ChIP-seq analysis revealed strong H3K27me3 enrichment at both loci in ACR10, correlating with transcriptional silencing. In contrast, H3K27me3 was undetectable at the *Avr3a* locus in P6497 and at either locus in sc10, consistent with their transcriptional activation (Fig. 1D and E). Together, these findings establish H3K27me3 as a key determinant of *Avr1b* and *Avr3a* silencing and suggest that epigenetic silencing of effector genes contributes to strain-specific virulence in a host-dependent manner.

### Abolished H3K27me3 and released expression of *Avr* genes in *PsSu(z)12-*edited mutant activates effector-triggered immunity

**Figure 2.**
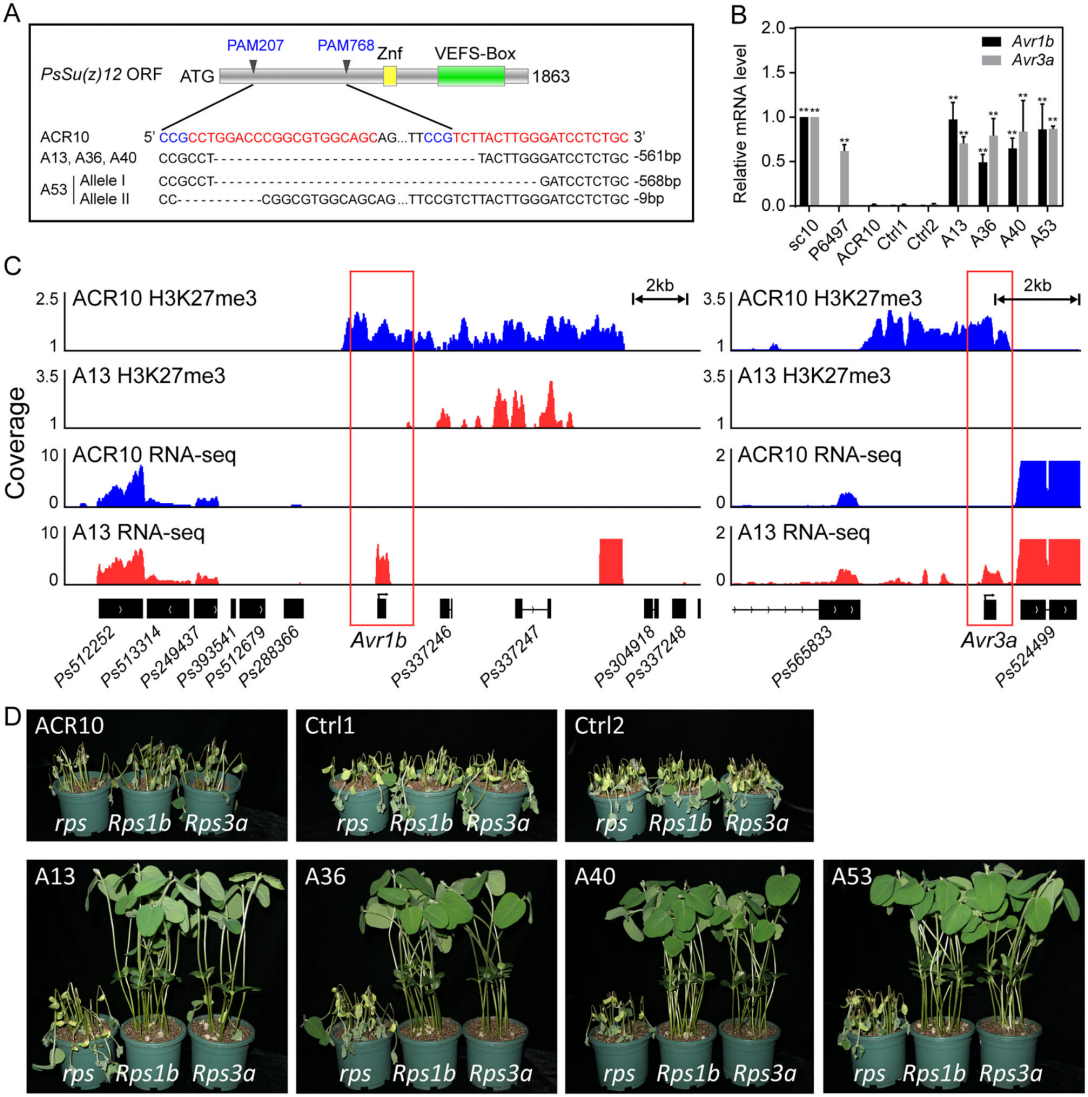
Editing of the *PsSu(z)12* locus in ACR10 released the expression of *Avr1b* and *Avr3a* and reduced the virulence. (**A**) The *PsSu(z)12* locus was edited using CRISPR/Cas9. A13, A36, and A40 are homozygous mutants with a 561-bp deletion. A53 is a heterozygous mutant, with one allele carrying a 561-bp deletion and the other allele carrying a 9-bp deletion. The positions of the sgRNAs are indicated by blue protospacer adjacent motif (PAM) sequences and red target sequences. (**B**) Relative mRNA levels of *Avr1b* and *Avr3a* in the mycelia of *PsSu(z)12* mutants measured by RT-qPCR. Transcript levels were normalized to *Actin* as the internal standard and presented relative to sc10 (set as 1). Data are shown as mean ± SEM from three biological replicates (** indicates *P* < .01, compared to ACR10, student’s *t*-test). (**C**) H3K27me3 ChIP-seq and RNA-seq data from the mycelia stage of ACR10 and A13 for the *Avr1b* and *Avr3a* loci are visualized in the IGV browser. H3K27me3 coverage was normalized to input as FE using MACS3, representing the mean value of three biological replicates. The coverage of RNA-seq reads was normalized using BPM and merged as the mean value of two biological replicates. (**D**) Virulence assays of control and mutant strains on soybean cultivars *Williams* (*rps*), *L77-1863* (*Rps1b*), and *L83-570* (*Rps3a*). Phenotypes were photographed 3 days after hypocotyl inoculation. Three biological replicates were performed with similar results.

Because ACR10 uniquely exhibits simultaneous silencing of both *Avr1b* and *Avr3a*, it provides an ideal background to test whether H3K27me3 regulates multiple *Avr* effectors in a strain-dependent manner. *PsSu(z)12* encodes a core component of the PRC2 complex responsible for catalyzing H3K27me3. To assess the role of H3K27me3 in the silencing of both *Avr1b* and *Avr3a*, we generated *PsSu(z)12* mutants in ACR10 using CRISPR/Cas9 technology with two guide RNAs (sgRNA207 and sgRNA768). Four independent mutants (A13, A36, A40, and A53) were isolated. Among them, A13, A36, and A40 harbored a 561-bp in-frame deletion in the *PsSu(z)12* coding sequence, while mutant A53 carried a heterozygous mutation with one allele containing a 561-bp deletion and the other a 9-bp in-frame deletion (Fig. 2A and Supplementary Fig. S3A). Additionally, we recovered two control strains, Ctrl1 and Ctrl2, in which the *PsSu(z)12* locus was not edited, from the same transformation experiment. Consistent with our previous findings on *PsSu(z)12* mutants in the P6497 background [14], these mutants did not show any defects *in vitro* growth on regular V8 media (Supplementary Fig. S3B and C). SMART domain prediction [57] of the 561-bp deletion revealed only a short low-complexity region (15 amino acids) and no confidently assigned conserved domains; however, we cannot exclude that it affects an uncharacterized but essential region required for PRC2 activity.

Subsequently, RT-qPCR analysis was performed to evaluate *Avr1b* and *Avr3a* expression across the examined strains (Fig. 2B). The expression of *Avr1b* and *Avr3a* was released in all four ACR10 *PsSu(z)12* mutants, in contrast to their silenced state in the wild-type strain. Next, we selected A13 as a representative mutant for ChIP-seq and RNA-seq to assess H3K27me3 occupancy and gene expression at the *Avr1b* and *Avr3a* loci (Fig. 2C). ChIP-seq revealed a complete loss of H3K27me3 at both loci in A13, resembling the pattern observed at *Avr1b* in T34, a *PsSu(z)12* mutant derived from the *Avr1b*-silenced strain P6497 [14] (Supplementary Fig. S4). Consistently, RNA-seq confirmed transcriptional reactivation at both loci. Integration of gene expression, histone modification, and phenotypic data from three natural strains (ACR10, P6497, and sc10) and mutants from two genetic backgrounds (A13 and T34) revealed that H3K27me3 modifications are key determinants of *Avr1b* and *Avr3a* gene expression, thereby shaping the virulence phenotype of *P. sojae* (Figs 1D and 2C and Supplementary Fig. S4).

Based on the avirulence activity of *Avr1b* and *Avr3a*, we assessed the pathogenicity of mutants (A13, A36, A40, and A53) in ACR10 background on soybean plants carrying the *Rps1b* and *Rps3a* resistance genes. As expected, all four mutants lost their virulence on *Rps1b-* and *Rps3a*-carrying plants but remained fully virulent on the susceptible cultivar *Williams* (Fig. 2D). These results suggested that the loss of *PsSu(z)12* disrupted H3K27me3-mediated silencing of *Avr1b* and *Avr3a*. The reactivation of these genes allowed their recognition by *Rps1b* and *Rps3a* to trigger immunity, resulting in a loss of virulence. This finding highlights the crucial role of *PsSu(z)12* in epigenetically regulating effector gene expression to evade host immune responses.

### *PsSu(z)12* complementation differentially restores H3K27me3 and silencing of *Avr1b* and *Avr3a*

**Figure 3.**
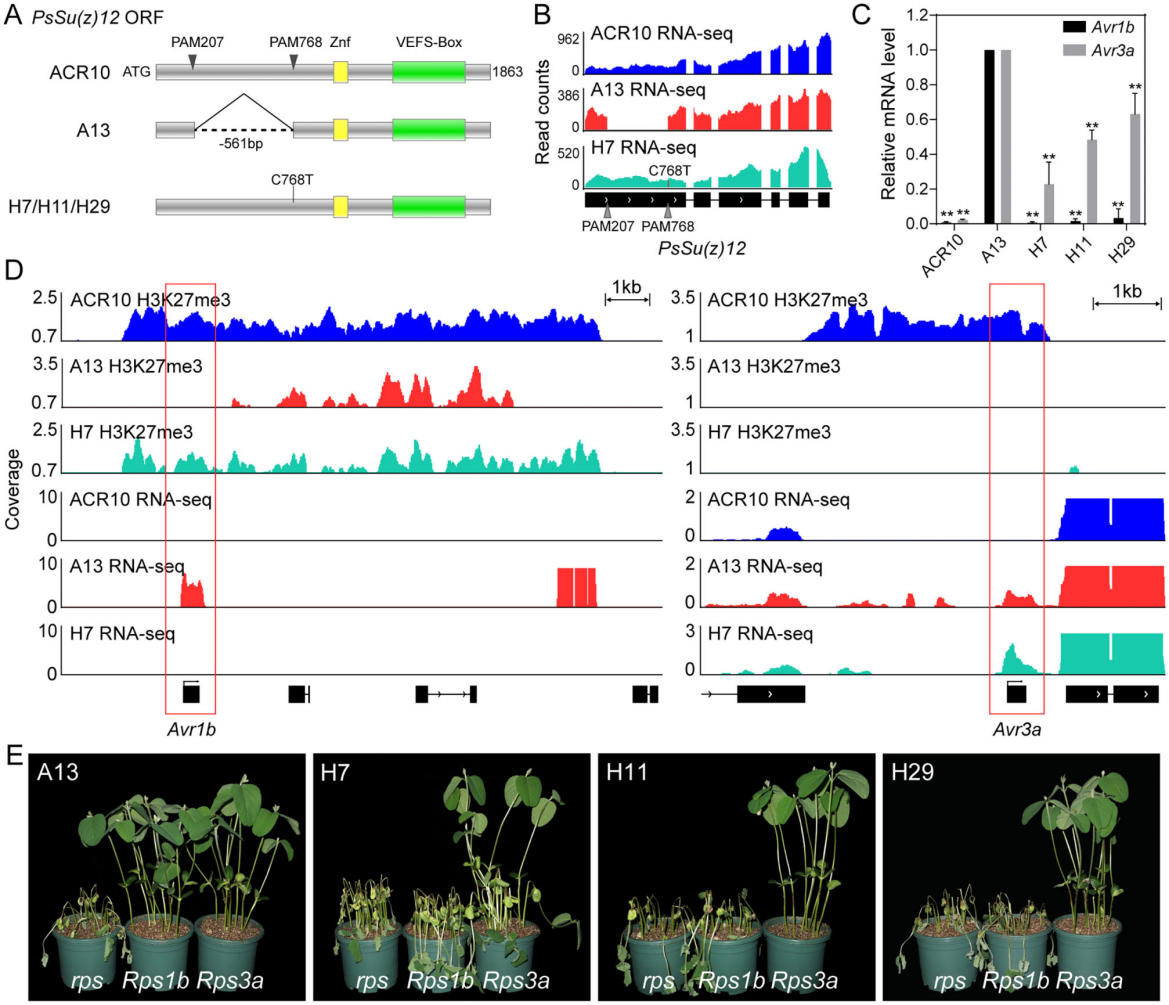
*PsSu(z)12*-complemented strains exhibit partially rescued virulence and different recovery of silencing marks at the *Avr1b* and *Avr3a* loci. (**A**) Schematic representation of the *PsSu(z)12* open reading frame in ACR10, A13, and complemented mutants (H7, H11, H29). The locations of PAM sites (PAM207 and PAM768), the zinc finger domain, and VEFS-box are indicated. Compared to the wild-type ACR10, the A13 mutant contains a 561-bp in-frame deletion, whereas the complemented lines (H7, H11, and H29) harbor a synonymous point mutation (C768T) that disrupts the PAM site without altering the amino acid sequence. (**B**) IGV visualization of RNA-seq alignments (displayed as unnormalized read counts from BAM files) showing *PsSu(z)12* expression in ACR10, A13, and complemented strain H7. The 561-bp deletion in A13 results in a clear loss of transcript coverage across the deleted region. In H7, expression is restored, including the presence of the synonymous C768T mutation. (**C**) Relative mRNA levels of *Avr1b* and *Avr3a* in ACR10, A13, and complemented strains (H7, H11, and H29), measured by RT-qPCR. Transcript levels were normalized to *Actin* as an internal control. A13 served as the positive (expressed) control. Data represent mean ± SEM from three biological replicates (*P* < .01, compared to ACR10, Student’s *t*-test). (**D**) H3K27me3 ChIP-seq (FE, mean of three replicates) and RNA-seq (BPM, mean of two replicates) profiles were visualized in the IGV browser for the *Avr1b* and *Avr3a* loci in ACR10, A13, and H7 strains. Boxes highlight the *Avr1b* and *Avr3a* loci. (**E**) Virulence assays of A13 and complemented mutants (H7, H11, and H29) on soybean cultivars *Williams* (*rps*), *L77-1863* (*Rps1b*), and *L83-570* (*Rps3a*). Images were taken 3 days after hypocotyl inoculation, showing differences in virulence among the strains.

To further validate the role of *PsSu(z)12* in H3K27me3-mediated gene silencing, we performed *in situ* complementation of the A13 mutant using a gRNA-mediated HDR strategy (Supplementary Fig. S5; see the “Materials and methods” section) [41]. The donor construct contained the *PsSu(z)12* coding region deleted in A13, flanked by 1-kb homologous arms, and introduced a synonymous C768T substitution as a diagnostic marker for complementation. Three independent complemented strains (H7, H11, and H29) were obtained (Fig. 3A and Supplementary Fig. S5). Sanger sequencing confirmed precise restoration of the *PsSu(z)12* open reading frame along with the C768T marker. RNA-seq further verified the recovery of full-length transcript (Fig. 3B and Supplementary Fig. S5). Notably, re-cleavage was not detected, as evidenced by stable sequence integrity in both Sanger sequencing and RNA-seq. These results demonstrate successful genetic complementation of the A13 mutant.

However, RT-qPCR analysis revealed differential transcriptional outcomes of *Avr1b* and *Avr3a* in the complemented strains (Fig. 3C). *Avr1b* was completely silenced in all three strains, whereas *Avr3a* remained transcriptionally active, albeit reduced, showing 22.8% to 63.2% downregulation relative to wild-type ACR10. To further examine the chromatin basis of these changes, we performed ChIP-seq and RNA-seq on the representative mutant A13 and complemented strain H7. These analyses revealed re-establishment of H3K27me3 at the *Avr1b* locus in H7, consistent with transcriptional re-silencing, while no H3K27me3 enrichment was detected at the *Avr3a* locus (Fig. 3D).

Consistent with these molecular profiles, virulence assays on soybean plants carrying *Rps1b* or *Rps3a* revealed differential restoration of infection ability (Fig. 3E). All three complemented strains regained full virulence on *Rps1b* plants, comparable to wild-type ACR10. By contrast, only H7 exhibited partial recovery on *Rps3a* plants, with intermediate virulence (52.5% lethality across five replicates), whereas H11 and H29 remained avirulent. These results indicate that *PsSu(z)12* complementation restores H3K27me3 and gene silencing at *Avr1b*, enabling immune evasion on *Rps1b* plants. In contrast, the failure to restore full silencing and virulence at *Avr3a* suggests that *PsSu(z)12* alone is insufficient to re-establish H3K27me3 at this locus, pointing to locus-specific differences in regulatory requirements.

These observations prompted us to examine the sRNA profiles at *Avr1b* and *Avr3a*, as another major epigenetic pathway for gene silencing, to assess whether sRNA accumulation contributes to the observed differences in transcriptional regulation and virulence phenotypes.

### sRNAs at the *Avr1b* and *Avr3a* loci coordinate with H3K27me3 and potentially underlie contrasting complementation outcomes

**Figure 4.**
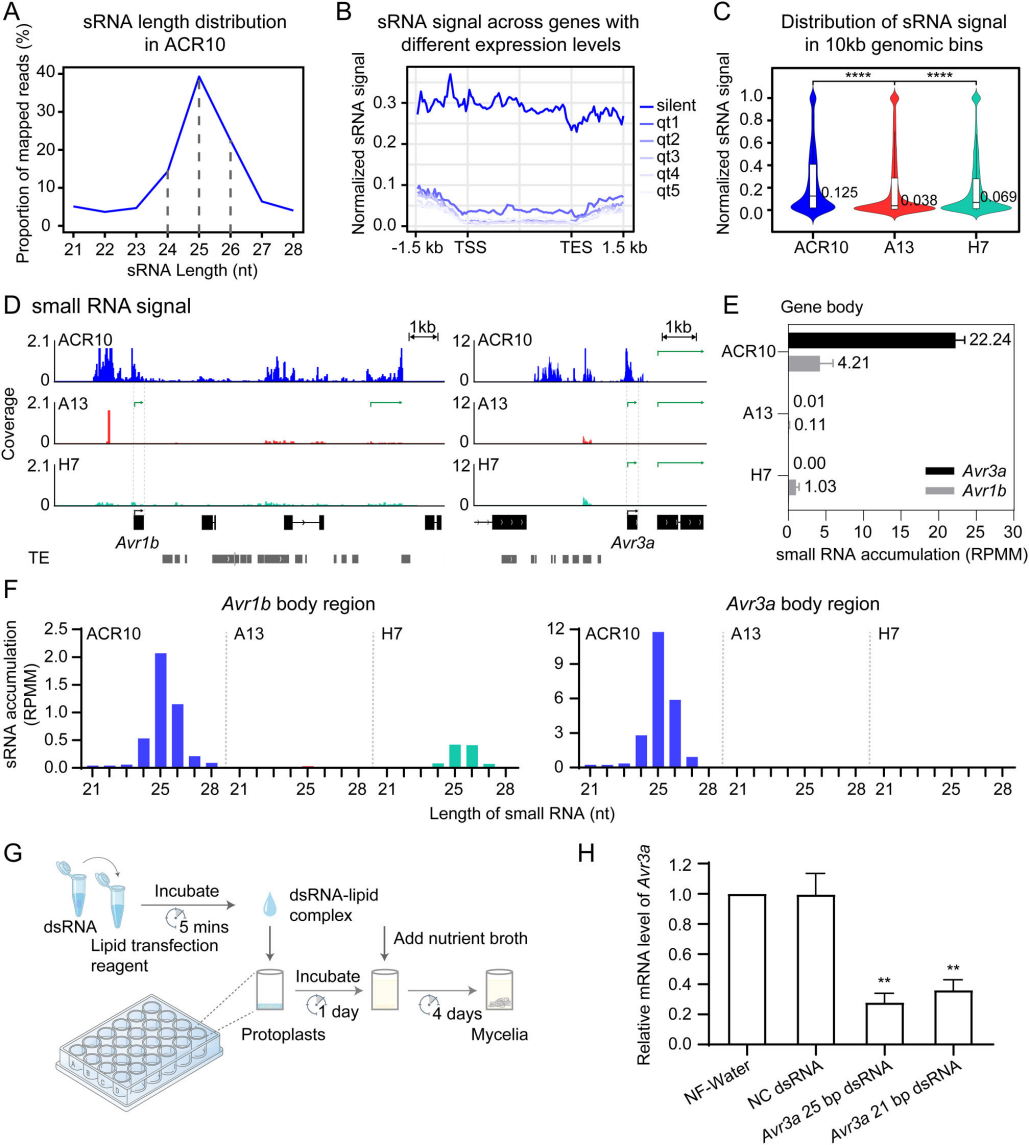
sRNA features and locus-specific accumulation *PsSu(z)12*-edited strains. (**A**) Length distribution of sRNAs in wild-type ACR10. (**B**) Normalized sRNA signals (CPM, counts per million mapped) across genes grouped by transcriptional levels. Genes with TPM <0.1 were classified as “silent”; expressed genes were divided into five equal-sized bins (qt1–qt5) from low to high expression. TSS: transcription start site; TES: transcription end site. (**C**) Violin and box plots showing genome-wide sRNA abundance (normalized per 10-kb bin) across ACR10, A13, and H7. sRNA signals were Min–Max normalized to a 0–1 range for cross-strain comparison. Median values are indicated. Statistical significance was assessed by Kruskal–Wallis test followed by post hoc Wilcoxon rank-sum tests. ^****^ denotes *P* < .0001. (**D**) Genome browser view of sRNA coverage (CPM, mean of biological replicates) at the *Avr1b* and *Avr3a* loci: ACR10 and A13 (*n* = 3); H7 (*n* = 2). Line arrows mark active gene transcription in specific strains. TE predictions are shown below. (**E**) Accumulation of sRNAs (RPMM) across the gene bodies of *Avr1b* and *Avr3a* in the indicated strains. (**F**) Length distribution of sRNAs mapped to the *Avr1b* and *Avr3a* gene bodies in ACR10, A13, and H7. *x*-axis: sRNA length (nt); *y*-axis: RPMM. (**G**) Schematic of lipid-mediated dsRNA transfection into *P. sojae* protoplasts. Protoplasts were incubated with dsRNA–lipid complexes for one day, followed by four days of culture in nutrient broth to allow mycelial growth. (**H**) RT-qPCR analysis of *Avr3a* expression in strain A13 after transfection with synthetic dsRNAs. The 25-bp dsRNA targets a region starting 79 bp downstream of the *Avr3a* start codon, while the 21-bp dsRNA overlaps this site, starting at +83 bp. The NC dsRNA was generated by randomly shuffling the 25-bp sequence to ensure no genomic targets. Data are presented as mean ± SD from three biological replicates (*n* = 3). Asterisks (**) indicate a significant difference compared to the NF-water control (*P* < .01, one-way ANOVA).

To investigate whether sRNAs contribute to effector gene silencing, we profiled sRNAs at the mycelial stage using wild-type strains, representative mutants, and a complemented strain. sRNA profiling of ACR10 revealed a predominant enrichment of 24–26 nt sRNAs, which accounted for 76% of the total mapped reads (Fig. 4A). Genome-wide comparison across genes with distinct transcriptional states showed that silenced genes exhibited the highest sRNA coverage, whereas actively expressed genes had only background levels (Fig. 4B). A similar pattern was observed for H3K27me3, which was enriched at silenced genes and depleted at expressed genes (Supplementary Fig. S6A). Further stratification by length revealed that 21–27 nt sRNAs were strongly associated with gene silencing, while 28 nt sRNAs showed much weaker correlation (Supplementary Fig. S7A).

To assess the global impact of *PsSu(z)12* disruption on sRNA abundance, we quantified normalized sRNA signals in 10-kb genomic bins. Compared to wild-type ACR10, genome-wide sRNA accumulation was markedly reduced in the *PsSu(z)12* mutant A13 and partially restored in the complemented strain H7 (Fig. 4C). This global pattern paralleled H3K27me3 dynamics: levels of the repressive mark were diminished in A13 and partially recovered in H7 (Supplementary Fig. S6B). In contrast, sRNA size distribution was unaffected, with all strains showing dominant enrichment of 24–26 nt sRNAs (Supplementary Fig. S7B). These results indicate that *PsSu(z)12* is required for maintaining sRNA abundance genome-wide but does not affect sRNA size profiles.

Locus-specific analyses revealed distinct differences at the *Avr1b* and *Avr3a* loci (Fig. 4D and E). In ACR10, sRNA accumulated over both coding regions. In mutant A13, both sRNA signals and H3K27me3 marks were abolished, coinciding with gene reactivation. In complemented strain H7, sRNAs were partially restored at the *Avr1b* locus, along with H3K27me3 re-establishment. However, neither sRNA restoration nor H3K27me3 recovery was detected at the *Avr3a* locus, indicating a tight coupling between sRNA accumulation and H3K27me3 deposition at effector loci. This correlation was further supported by the patterns in P6497 and its *PsSu(z)12* mutant T34: both sRNAs and H3K27me3 were present at the *Avr1b* locus in P6497 but absent in T34. In contrast, in wild-type strain sc10, where both *Avr* genes were actively expressed, neither sRNAs nor H3K27me3 were detected at either locus (Supplementary Fig. S8A and B). Together, these results demonstrate that sRNA accumulation is coordinated with H3K27me3 deposition at effector loci and supports a dual-layer epigenetic mechanism underlying *Avr* gene silencing. However, the differential recovery of H3K27me3 and sRNA levels at the *Avr1b* and *Avr3a* loci likely underlies their divergent complementation outcomes, reflecting locus-specific epigenetic plasticity potentially shaped by sRNA dynamics.

To further investigate the differential complementation outcomes of *Avr1b* and *Avr3a*, we analyzed sRNA characteristics at each locus (Fig. 4D–F and Supplementary Fig. S8). At the *Avr1b* locus, silenced strain ACR10 accumulated 24–26 nt sRNAs across the promoter and gene body. However, upon activation in A13, the promoter sRNA profile shifted toward shorter 21 to 22 nt species with a sharply focused distribution (Fig. 4D and Supplementary Fig. S8C). Notably, in H7, even a partial recovery of sRNAs (1.03 RPMM, with 25 to 26 nt dominating), in concert with H3K27me3, was sufficient to maintain *Avr1b* silencing. In contrast, at the *Avr3a* locus, ACR10 also exhibited a dominance of 24–26 nt sRNAs, but at an abundance ~ 5-fold higher than that at *Avr1b*, reflecting a greater reliance on sRNA-mediated regulation. When *Avr3a* was activated in A13, gene body-associated sRNAs were completely eliminated, with only residual 24–26 nt sRNAs persisting at a TE-proximal region. These sRNAs failed to be re-established in H7, coinciding with the failure to re-silence the gene. Together, these results reveal locus-specific differences in sRNA length, abundance, and TE association, corresponding to the divergent complementation outcomes of *Avr1b* and *Avr3a*.

Since sRNA accumulation was not restored at the *Avr3a* locus upon *PsSu(z)12* complementation, we next assessed whether *Avr3a*-targeting sRNAs are sufficient to trigger gene silencing. Based on the ACR10 sRNA-seq dataset, we selected a highly abundant 25 nt sRNA and a partially overlapping 21 nt sRNA to synthesize corresponding dsRNAs. Using a protoplast-mediated transformation strategy, these dsRNAs were introduced into the *PsSu(z)12* mutant A13, which exhibits reactivated *Avr3a* expression and lacks endogenous sRNAs at this locus (Fig. 4G). RT-qPCR analysis revealed a significant reduction of *Avr3a* transcript levels in regenerated mycelia following *Avr3a*-targeting dsRNA treatment, compared to the NF-water and NC dsRNA treatments (Fig. 4H). These results demonstrate that *Avr3a*-targeting sRNA precursors are sufficient to suppress *Avr3a* expression under conditions where H3K27me3 deposition is disrupted, supporting a direct role for sRNA-mediated repression of effector genes.

### Identification of H3K27me3-regulated RxLR effector genes in *P. sojae*

To explore whether the H3K27me3 regulatory mechanism applies to other RxLR effector genes, we performed a comprehensive genome-wide analysis integrating H3K27me3-enriched ChIP-seq and RNA-seq across wild-type ACR10, the *PsSu(z)12* mutant A13, and complemented strain H7.

**Figure 5.**
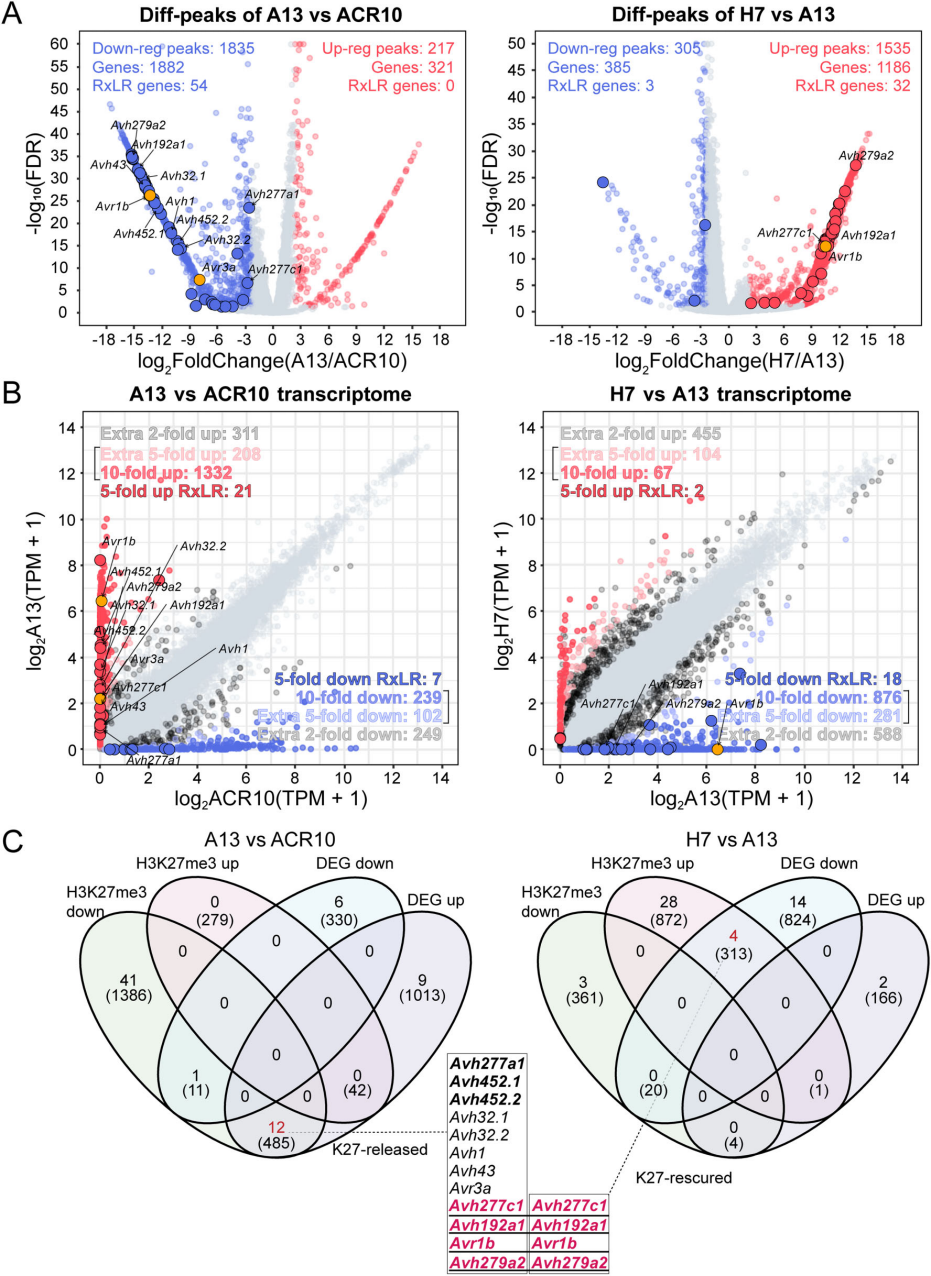
Integration of H3K27me3 and transcriptome data from ACR10, A13, and H7 strains identifies a group of RxLR effector genes strongly associated with H3K27me3. (**A**) Volcano plots showing H3K27me3 five-fold differentially modified peaks between A13 and ACR10 (left) and between H7 and A13 (right). H3K27me3 differentially modified RxLR effector genes are highlighted, and those screened in panel (C) are denoted, with *Avr* genes specifically indicated. (**B**) Scatter plots of transcriptome comparisons between A13 and ACR10 (left) and H7 and A13 (right). Gene expression is plotted as log_2_(TPM + 1). Genes with >5-fold upregulation or downregulation are highlighted. RxLR effector genes screened in panel (C) are denoted, with *Avr* genes specifically indicated. (**C**) Venn diagrams showing overlap between genes with five-fold H3K27me3 DMGs and five-fold DEGs in A13 versus ACR10 (left) and H7 versus A13 (right). The numbers indicate the corresponding RxLR effector genes, with the numbers in parentheses representing the total count of genes in each category. RxLR effector genes classified as K27-released (H3K27me3 downregulated with transcriptional upregulation in A13 compared to ACR10) and K27-rescued (H3K27me3 upregulated with transcriptional downregulation in H7 compared to A13) are listed below, with transcriptional differentially downregulated genes in H7 bolded.

For H3K27me3 ChIP-seq analysis, differential peaks between ACR10 and A13 were identified using DiffBind, applying a threshold of ≥5-fold change and an adjusted *P*-value <.05. A total of 5114 consensus peaks were defined across both datasets, among which 1835 were significantly downregulated and 217 were upregulated in A13 compared to ACR10. Using ChIPseeker annotation and Bedtools intersect, these peaks were associated with 1882 and 321 genes, respectively, including 54 RxLR effector genes showed less enrichment and none had more enrichment. Using the same analytical approach, we performed H3K27me3 differential peak analysis between H7 and A13. This analysis identified 1535 upregulated peaks and 305 downregulated peaks. Subsequent annotation and intersection associated these peaks with 1186 and 385 genes, respectively. Among these, 32 RxLR effector genes were linked to upregulated peaks, while three RxLR effector genes were linked to downregulated peaks (Fig. 5A). These results are consistent with the expected role of *PsSu(z)12* in maintaining H3K27me3-mediated epigenetic silencing. In A13, H3K27me3 modifications are abolished in a substantial number of RxLR genes, with a subset restored in H7. Notably, we observed H3K27me3 downregulation on both *Avr1b* and *Avr3a* loci in A13, but only see upregulation on *Avr1b* in H7 compared with A13 (Fig. 5A).

Subsequently, we analyzed DEGs between ACR10 and A13 using DESeq2 with a *P*-value <.05. In the result we identified 1540 genes with >5-fold upregulation, including 1332 showing over 10-fold upregulation. Conversely, 341 genes were downregulated with >5-fold, including 239 with over 10-fold downregulation. Similarly, in the comparison between H7 and A13, 1157 genes were downregulated by >5-fold, including 876 with over 10-fold downregulation, while 385 genes were upregulated by >5-fold, with 67 showing over 10-fold upregulation (Fig. 5B). These results are consistent with the expected changes of gene expression following the variation of H3K27me3 modifications in *PsSu(z)12* editing and complemented strains. In A13, a large number of genes showed diminished H3K27me3 modifications, leading to widespread transcriptional activation, as observed for *Avr1b* and *Avr3a*. In the complemented mutants, partial reestablishment of H3K27me3 modifications reflected the re-silencing of a subset of these genes, including *Avr1b*. Genome-wide annotation of H3K27me3 peaks in ACR10 (Supplementary Fig. S6C) revealed that most peaks were located within gene bodies and promoter regions, while a smaller fraction overlapped with intergenic regions and TEs. The majority of differential peaks identified in both ACR10 versus A13 and A13 versus H7 comparisons were also associated with gene body and promoter regions, indicating that *PsSu(z)12* disruption predominantly affects H3K27me3 occupancy at gene-associated loci.

Next, a combined analysis between H3K27me3 differentially modified genes (DMGs) and DEGs in both ACR10 versus A13 and A13 versus H7 comparisons was performed (Fig. 5C). This analysis revealed that 12 RxLR effector genes exhibited reduced H3K27me3 and transcriptional upregulation in A13, indicating suppression by histone modification (Fig. 5C, left). These genes are classified as H3K27me3-released RxLR effector genes (K27-released RxLR). Among these, seven genes exhibiting re-silence in H7 (bolded in Fig. 5C list), including four with restored H3K27me3 deposition (K27-rescued). Applying the same approach, we identified seven K27-released RxLR in P6497, three of which overlapped with those identified in ACR10 (Supplementary Fig. S9). In summary, we identified a total of 16 unique RxLR effector genes that are specifically regulated by *PsSu(z)12*-dependent H3K27me3 across the two isolates, indicating that H3K27me3-mediated gene silencing is commonly employed by *P. sojae* strains.

### sRNAs act in concert with H3K27me3 to regulate RxLR effector genes expression in a *PsSu(z)12-*dependent manner

**Figure 6.**
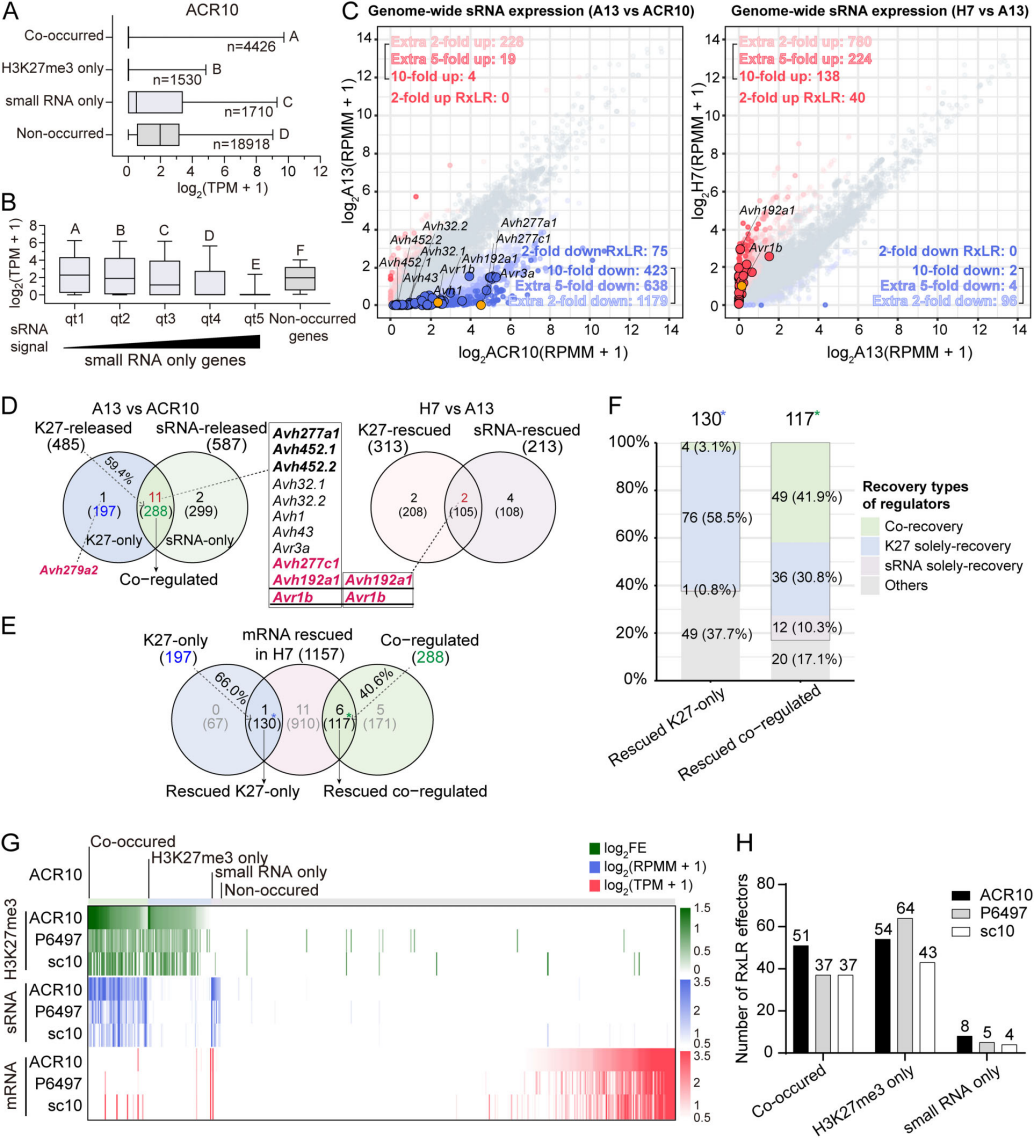
Genome-wide analysis of H3K27me3 and sRNA reveals a co-occurrence pattern regulating RxLR effector gene expression. (**A**) Boxplots showing the average expression levels [log_2_(TPM + 1)] in ACR10, grouped by regulatory category: co-occurrence of H3K27me3 and sRNA (co-occurred, mean FE ≥1 and RPMM ≥1), H3K27me3 only (mean FE ≥1 and RPMM <1), small RNA only (mean FE <1 and RPMM ≥1), and non-occurred genes (mean FE <1 and RPMM <1). Gene counts per group are indicated. Statistical differences were assessed using Kruskal–Wallis rank-sum tests. Error bars represent the minimum and maximum values. (**B**) Boxplots of gene expression across increasing sRNA abundance quartiles (q1–q5) for small RNA only genes in ACR10. Non-occurred genes are shown for comparison. Statistical differences were assessed using the Kruskal–Wallis one-way analysis of variance by ranks. Error bars represent the 10th and 90th percentiles. (**C**) Scatter plots comparing genome-wide sRNA abundance in A13 versus ACR10 (left) and H7 versus A13 (right). Values represent log_2_(RPMM + 1). Genes with > 2-fold upregulation or downregulation are highlighted; significantly changed RxLR genes are labeled. (**D**) Venn diagrams showing the overlap between K27-released and sRNA-released genes in A13 versus ACR10 (left) and K27-rescued and sRNA-rescued genes in H7 versus A13 (right). In the left panel, transcriptionally upregulated genes with downregulated H3K27me3 but no significant change in sRNAs are classified as K27-only, while genes with downregulated sRNAs and no significant changes in H3K27me3 are classified as sRNA-only. A total of 288 overlapped genes (59.4% of K27-released) are classified as co-regulated. RxLR genes in the overlaps are listed; those rescued in H7 are bolded. (**E**) Venn diagram showing overlap of K27-only and co-regulated genes (from D) with genes showing >5-fold mRNA downregulation in H7 compared to A13 (mRNA rescued). A total of 130 genes (66.0% of K27-only) were identified as re-silenced K27-only, and 117 genes (40.6% of co-regulated) were identified as rescued co-regulated. (**F**) Stacked bar chart elucidates the recovery types of regulators of the two subgroups of mRNA rescued genes in panel (E): rescued K27-only and rescued co-regulated. (**G**) Heatmap illustrating the polymorphic distribution of H3K27me3 and sRNAs in RxLR effectors among three natural strains. (**H**) Statistical analysis of the number of RxLR effectors corresponding to different regulatory types across three natural strains.

In above analysis, multiple RxLR DEGs lacked detectable H3K27me3 changes in ACR10 mutants, pointing to the involvement of additional regulatory layers. Given that sRNA has been implicated in silencing effectors in *P. sojae* [13, 27], we next performed a genome-wide analysis to investigate the interplay between these two regulatory layers in ACR10. We observed that 74.3% (4426/5956) of H3K27me3-modified genes co-occurred with sRNAs and 72.1% (4426/6136) of sRNA-producing genes co-occurred with H3K27me3, demonstrating a strong overlap between the two silencing pathways in *P. sojae* (Fig. 6A). Moreover, the result showed that genes marked by H3K27me3, either alone or co-occurring with sRNAs, were predominantly silenced. In contrast, genes associated solely with sRNAs exhibited reduced expression levels compared to genes without silencing marks, with a median TPM value of <1 (0.41), indicating partial silencing. These findings suggest that H3K27me3 serves as the primary determinant of transcriptional silencing, while sRNAs provide an additional layer of regulation, contributing to a more nuanced control of gene expression. This tight coordination is further illustrated by a circular genome map showing extensive overlap between H3K27me3 and sRNA reads in silenced regions (Supplementary Fig. S10). A length comparison of sRNAs between the “co-occurred” group and the “small RNA only” group revealed no significant differences (Supplementary Fig. S11). However, when “small RNA only” genes were ranked by sRNA abundance, a positive correlation with transcriptional silencing emerged (Fig. 6B). This suggests that sRNAs alone can mediate gene repression in a dose-dependent manner, consistent with a canonical RNAi mechanism, and is further supported by the synthetic dsRNA transfection assay (Fig. 4H). Collectively, these results indicate that H3K27me3 maintains a stable transcriptional off state with or without accompanying sRNAs, indicating a TGS mode or a combination of TGS and PTGS, whereas sRNAs alone function in a quantitative manner resembling PTGS. These two types of regulators act in concert to fulfill distinct requirements for gene repression.

Next, we compared genome-wide sRNA profiles between A13 and ACR10 and identified 2240 loci with a two-fold reduction in sRNA levels in A13 (adjusted *P* < .05), highlighting 75 RxLR effector genes including *Avr1b* and *Avr3a*. This reduction mirrored the downregulation pattern of H3K27me3. Conversely, only 251 loci showed a two-fold increase in sRNA accumulation (Fig. 6C, left). In H7, 1142 genes exhibited a two-fold increase in sRNA levels compared to A13, including 40 RxLR effector genes like *Avr1b*. This increase aligned with the re-establishment of H3K27me3 (Fig. 6C, right). Notably, a similar reduction of sRNAs was also revealed in the independent T34 mutant compared to P6497, further supporting this conclusion (Supplementary Fig. S12). These findings highlight the strong association between sRNAs and H3K27me3, which collectively coordinate gene silencing via a *PsSu(z)12*-mediated pathway.

Analogous to the identification of K27-released and K27-rescued genes in Fig. 6C, we identified 587 sRNA-released genes (including 13 RxLR effector genes) and 213 sRNA-rescued genes (including six RxLR effector genes) (Supplementary Fig. S13A). The sRNA-released genes were characterized by a reduction in sRNA levels, alongside an upregulation of gene expression in the A13 mutant relative to the ACR10 wild-type strain. Conversely, the sRNA-rescued genes exhibited restored sRNA levels in H7, accompanied by transcriptional repression. These data indicate that sRNAs are predominantly involved in the gene regulation in *P. sojae*.

To systematically investigate the interplay between H3K27me3 and sRNAs, we performed a comparative analysis of K27-released and sRNA-released genes (Fig. 6D left). The analysis revealed that 197 genes were activated exclusively by H3K27me3 depletion (K27-only), while 299 were activated solely by sRNA depletion (sRNA-only). Notably, the remaining 288 genes were co-regulated by both mechanisms (co-regulated), accounting for 59.4% of the K27-released genes. Strikingly, 11 out of 12 (91.7%) K27-released RxLR genes were co-regulated by sRNAs (Fig. 6D left and listed). The precise statistical values of H3K27me3, sRNA, and mRNA levels of these 12 RxLR genes were verified and are shown in Supplementary Fig. S14. As a further validation, a comparable proportion was observed in the comparison between T34 and P6497, where five out of seven K27-released RxLR genes were identified as co-regulated by sRNAs (Supplementary Fig. S12B and C). These findings underscore the substantial overlap between H3K27me3- and sRNA-mediated mechanisms in regulating gene expression, particularly in the silencing of critical effector genes.

To further investigate the role of *PsSu(z)12* in establishing silencing marks and regulating gene expression, we compared K27-rescued and sRNA-rescued genes in the complemented mutant H7 (Fig. 6D, right and listed). The analysis revealed that 208 genes were silenced exclusively by H3K27me3 complementation, while 108 were suppressed solely through recovered sRNAs. Notably, 105 out of 313 (33.5%) K27-rescued genes showed co-rescued sRNA accumulation, including two K27-released RxLR genes (Fig. 6D, right and listed). The partial rescue efficiency in H7 suggests that *PsSu(z)12* is sufficient for the establishment and maintenance of H3K27me3 and sRNAs at loci like *Avr1b* and *Avh192a1* but probably requires additional components for other RxLR genes.

Next, we investigated the transcriptional rescue efficiency of K27-released genes in the complemented strain H7, categorizing them based on the presence or absence of sRNA co-regulation. Venn diagram analysis revealed that 66.0% of K27-only genes (130 genes) regained silencing in H7, defining them as rescued K27-only genes. By comparison, only 40.6% of the co-regulated genes showed transcriptional rescue in H7 (rescued co-regulated genes) (Fig. 6E). Notably, the sole K27-only RxLR gene, *Avh279a2*, successfully restored silencing in H7. Among the 11 co-regulated RxLR effector genes, six demonstrated significant downregulation in H7 (bolded in Fig. 6D).

To investigate the mechanisms underlying transcriptional repression in complemented strains, we analyzed the recovery types of K27-only and co-regulated rescued genes in H7 (Fig. 6F). Among the 130 rescued K27-only genes, the majority (76, 58.5%) regained H3K27me3 deposition, while only a small fraction (4, 3.1%) showed concurrent recovery of both H3K27me3 and sRNAs. In contrast, of the 117 rescued co-regulated genes, a significantly larger fraction (49, 41.9%) displayed recovery of both regulators. The remaining genes in this group were primarily associated with either H3K27me3 alone (36, 30.8%) or sRNAs alone (12, 10.3%). These results indicate that H3K27me3 restoration is the primary correlate of gene re-silencing in H7, whereas sRNA recovery occurs at a subset of loci. Additionally, since all analyses were performed in the context of Su(z)12 complementation, sRNA-associated changes may reflect downstream or dependent effects of H3K27me3 recovery.

Furthermore, a number of genes in both categories were classified as “others,” indicating the involvement of additional mechanisms. This is further illustrated by the 194 rescued “sRNA-only” genes. Among these genes, only 33.0% were attributed to sRNA regulation alone, while 49.5% fell into the “others” category (Supplementary Fig. S13B). These findings suggest the restoration of silencing by *PsSu(z)12* at these loci is not solely dependent on sRNA recovery and likely involves other downstream or coordinated pathways.

Collectively, these data indicate that *PsSu(z)12* mediates gene silencing in *P. sojea* through multiple, layered mechanisms. H3K27me3 functions as the dominant regulatory mark, while sRNAs act as important collaborators that coordinate with H3K27me3. The subset of genes not explained by either mark underscores the contribution of additional, as yet unidentified, regulatory pathways.

### Divergent H3K27me3 and sRNA landscapes of RxLRs in natural *P. sojae* strains reveal adaptive strategy heterogeneity

Comparative analysis of H3K27me3 and sRNA distribution across 501 RxLR effector genes in three *P. sojae* strains (ACR10, P6497, and sc10) revealed both conserved principles and strain-specific regulatory architectures (Fig. 6G and H). A global inverse correlation between repressive epigenetic regulators and effector gene expression was evident across all strains by heatmap, indicating a shared silencing framework. However, the deployment of these regulators across strains varied substantially. ACR10 utilized a dual-layered silencing mechanism extensively, with the largest set of RxLRs co-occurred by H3K27me3 and sRNAs. P6497 instead showed the highest number of effectors controlled exclusively by H3K27me3, suggesting reduced reliance on sRNAs. Meanwhile, the sc10 strain exhibited the most sparsely regulated effector set, which may reflect more active effector expression or a reduced dependence on these two regulators. Notably, the sRNA-only class represented the smallest subset in all three strains, supporting the model that sRNAs primarily function in concert with H3K27me3 rather than as independent regulators of the RxLR repertoire.

Collectively, these findings uncover a strain-specific regulatory repertoire for RxLR effectors, shaped by distinct epigenetic architectures, that may underlie divergent host-adaptation strategies and pathogenic success.

## Discussion

**Figure 7.**
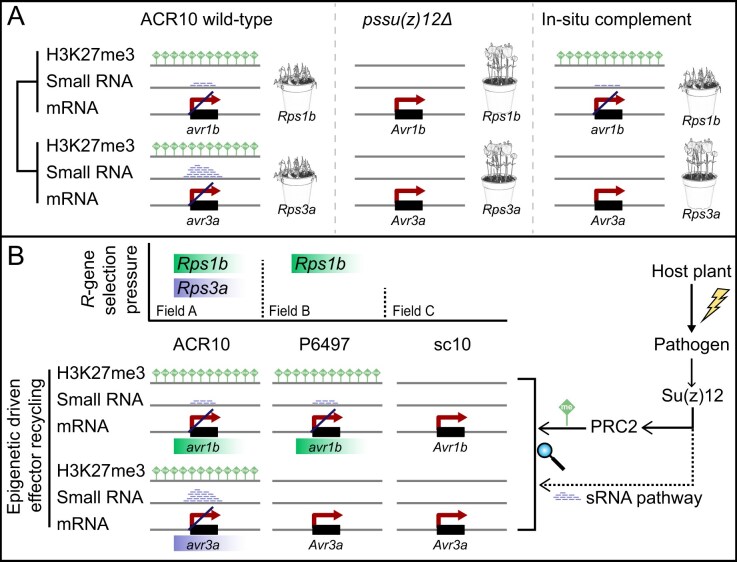
*PsSu(z)12*-dependent H3K27me3- and sRNA-mediated silencing of *Avr* genes enables immune evasion of *P. sojae*. (**A**) Locus-specific evidence from genetic analysis. In wild-type strain ACR10, both *Avr1b* and *Avr3a* are marked by H3K27me3 and associated sRNAs, resulting in transcriptional silencing and evasion of recognition by *Rps1b* and *Rps3a*, thereby showing virulent toward both plants. In *pssu(z)12Δ* mutants, loss of H3K27me3 deposition is accompanied by reduced sRNA accumulation, leading to the reactivation of *Avr1b* and *Avr3a* expression, thereby preventing the pathogen from escaping recognition by *Rps1b*- and *Rps3a*-carrying plants. In *in situ* complemented strains, restoration of H3K27me3 and sRNA on *Avr1b* locus re-establishes the silencing of *Avr1b* and restores virulence toward *Rps1b*-carrying plants. However, phenotypic complementation of *Avr3a* is not achieved, reflecting locus-specific differences in regulatory requirements. (**B**) Proposed model of dual-layered epigenetic control. In response to host-derived gene-for-gene pressure, pathogen coordinates two epigenetic silencing pathways through *PsSu(z)12*: PRC2-mediated deposition of H3K27me3 and the sRNA-associated pathway. These regulators act in concert to silence effector genes, providing a robust on/off switch that facilitates “effector recycling” under host immune surveillance and enhances adaptation.

Pathogens employ epigenetic mechanisms to silence effector genes (e.g. *Avr* genes), enabling evasion of plant immunity. In *P. sojae*, two major silencing mechanisms have been described: the deposition of the histone mark H3K27me3 by PRC2, which silences *Avr1b* in strain P6497 [14], and the transgenerational silencing of *Avr3a*, which is strongly correlated with the accumulation of sRNA [13, 58]. In this study, we show that *PsSu(z)12*, a core PRC2 subunit, integrates these silencing mechanisms at specific RxLR effector loci, including *Avr1b* and *Avr3a* (Fig. 7). Comparative analyses between wild-type and *PsSu(z)12*-edited mutants revealed that 11 out of 12 (91.7%) H3K27me3-regulated RxLR effectors were co-regulated by sRNAs, a rate far exceeding the genome-wide average (59.4%) (Fig. 6D). This enrichment highlights RxLR effectors as a hotspot for dual-layered silencing, providing a robust on/off regulatory switch that enables effector recycling under host immune surveillance and promotes pathogen adaptation.

Although Su(z)12 is traditionally viewed as a core PRC2 component, our data indicate that *PsSu(z)12* also contributes to sRNA accumulation, supporting its role as a regulatory hub linking H3K27me3-mediated TGS and sRNA-based regulation in *Phytophthora*. In plants, the RNA-directed DNA methylation (RdDM) mechanism establishes an indirect link between sRNAs and histone modifications through 5-methylcytosine (5mC)-mediated chromatin remodeling [59]. However, *Phytophthora* lacks detectable levels of 5mC methylation [60], raising questions about how these two regulatory layers interact in oomycetes. Intriguingly, analogous to the recruitment of H3K9 methylation SU(VAR)3–9 enzymes by sRNAs in plants, in *Paramecium tetraurelia*, Piwi-bound sRNAs recruit Ezl1/PRC2 via RING finger protein to TEs, suggesting a synergistic mechanism between PRC2 and the RNAi pathway in silencing TEs [61, 62]. In contrast, in *T. thermophila*, the EZL1 complex functionally couples nuclear RNAi with Polycomb-mediated heterochromatin formation and siRNA production [36]. In addition, Su(z)12 can bind nascent RNAs, potentially displacing PRC2 or facilitating RNA decay via associated ribonucleases [34, 35, 63, 64]. These findings point to a conserved logic of sRNA–Polycomb interactions across kingdoms. Based on these parallels, we propose two possible non-exclusive models to explain the coordination between sRNAs and H3K27me3 in *P. sojae*: (i) an sRNA-mediated recruitment model, in which sRNAs direct PRC2 to target loci to establish H3K27me3 and TGS, analogous to the RdDM pathway in plants; or (ii) a pre-mRNA-coupled recruitment model, in which low-level transcription generates nascent RNAs that both displace PRC2 to deposit H3K27me3 and simultaneously serve as sRNA substrates to facilitate decay, enabling flexible silencing in response to stimuli (a combined TGS/PTGS mechanism). Our genome-wide analysis reveals three regulatory modes by their epigenetic signatures (Supplementary Table S2). Genes marked exclusively by H3K27me3 (K27-only) exhibit robust transcriptional silencing, consistent with canonical TGS (Fig. 6A). In contrast, sRNA-only genes display dose-dependent repression, characteristic of PTGS (Fig. 6B). Co-occurred genes, which bear both H3K27me3 and sRNAs, are almost uniformly silenced, supporting the proposed integration of TGS and PTGS, consistent with either sRNA-mediated PRC2 recruitment or pre-mRNA-coupled recruitment.

Previous studies have suggested that while sRNAs are often necessary, they are not always sufficient for silencing, and that sRNA-mediated regulation can differ across strains [58]. Consistent with this, our analysis (Fig. 6G and H) showed that “small RNA only” categorized RxLR genes represent the smallest subset in all three natural strains, while both H3K27me3 and sRNA distributions exhibit substantial intra-strain locus specificity and inter-strain heterogeneity. In addition, the incomplete recovery of *Avr3a* silencing in the complemented strain may reflect differential locus requirements for Su(z)12. Although the deleted region lacks an annotated domain, it may be critical for PRC2 function at certain loci such as *Avr3a*. Collectively, these investigations indicate that H3K27me3 and sRNA act synergistically to reinforce repression in a context-dependent manner. Their regulatory role varies by strain and locus.

Integrating ChIP-seq and RNA-seq data identified 12 H3K27me3-regulated RxLR effector genes that showed simultaneous H3K27me3 loss and transcriptional activation following *PsSu(z)12* editing. These genes constitute 11.4% of the 105 H3K27me3-modified RxLR effector genes in ACR10. This proportion may be limited by the in-frame deletion of *PsSu(z)12*. However, achieving a complete knockout remains challenging due to potential lethality [14]. Similarly, in the circular genome map, only a limited number of regions exhibit alterations in H3K27me3 or sRNA in A13 or H7 (Supplementary Fig. S10). We propose that additional H3K27me3-regulated RxLR effector genes could be identified by editing other components of PRC2 or by investigating H3K27me3 modification differences across a broader range of *P. sojae* strains. Intriguingly, a subset of reactivated genes in strain H7 lacked restored H3K27me3 or sRNAs, suggesting the involvement of additional regulators (Fig. 6 E and F). Previous studies in *P. infestans* have shown that histone deacetylation can influence RNAi-mediated silencing [22, 65]. Recent findings demonstrated that H4K16ac, mediated by PsDMAP1/PsTIP60, aids adaptation of *P. sojae* to host-induced reactive oxygen species stress, underscoring acetylation’s role in virulence [66]. These observations suggest that other histone modifications, particularly acetylation, may offer deeper insights into the integrated regulation of gene expression and pathogen adaptation.

In summary, RxLR effector genes exhibit transcriptional polymorphism across natural *P. sojae* strains. *PsSu(z)12* coordinates H3K27me3 and sRNA-mediated silencing at key effector loci, establishing a dual-layered, reversible epigenetic switch that facilitates immune evasion and environmental adaptation (Fig. 7). As a central regulator of effector silencing, *PsSu(z)12* represents a promising molecular target for disrupting effector gene silencing in *P. sojae*. A deeper understanding of these pathogen adaptation mechanisms can inform the development of effective countermeasures, such as epigenetic inhibitors or dsRNA-based precision agriculture strategies.

## Supplementary Material

gkaf1426_Supplemental_File

## Data Availability

The RNA-seq dataset, ChIP-seq dataset, and sRNA-seq dataset produced during this study were deposited in the Sequence Read Archive (SRA) under BioProject Number PRJNA1206799. The RNA-seq data for P6497 and T34 were accessed from NCBI under accession numbers GSE116089 and GSE127207. The ChIP-seq data for P6497 and T34 were accessed from NCBI under accession number GSE127206. The sRNA-seq data for ACR10 were accessed from NCBI Bioproject PRJNA300858. The BigWig files for IGV visualization were archived in Zenodo at DOI: 10.5281/zenodo.17542274, which preserves a snapshot of the corresponding GitHub repository (https://github.com/LiyuanW21/P.sojae3.0), and the pre-configured genome browser session is available via the IGV website (https://tinyurl.com/3uj9j7yu).
