# Supplementary material for: Effector gene silencing coordinated by histone methylation and small RNAs enhances host adaptation in a plant pathogen
Source: Nucleic Acids Res. 2026 Jan 8;54(1):gkaf1426. doi: 10.1093/nar/gkaf1426 (PMC12781887; doi:10.1093/nar/gkaf1426)
Supplement: gkaf1426_Supplemental_File [file gkaf1426_supplemental_file.pdf]

Supplementary Figures

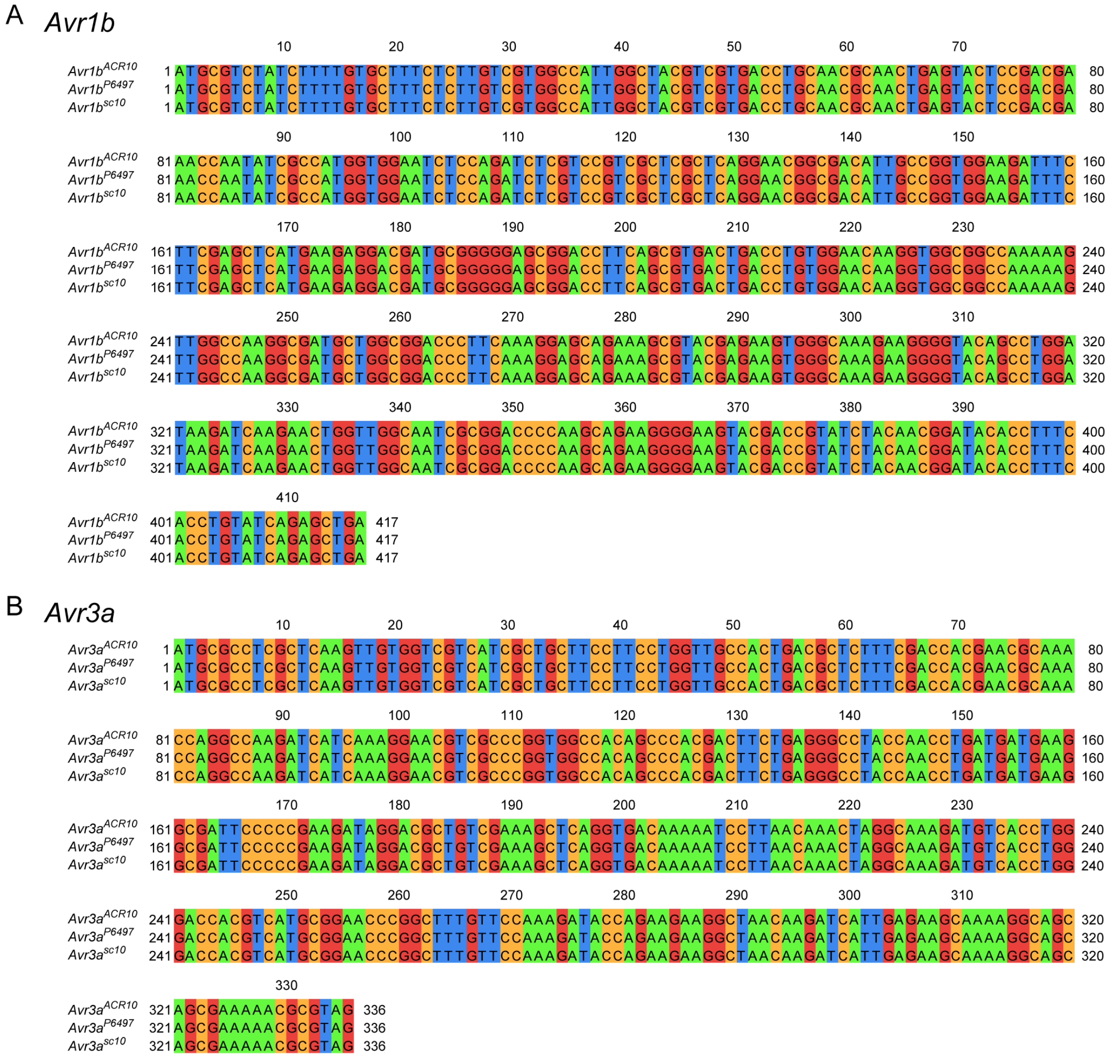

Figure S1. The coding sequences of *Avr1b* and *Avr3a* are respectively identical across strains. Multiple alignment of the *Avr1b* (A) *and Avr3a* (B) coding region nucleotide sequences from strains ACR10, P6497 and sc10 are shown.

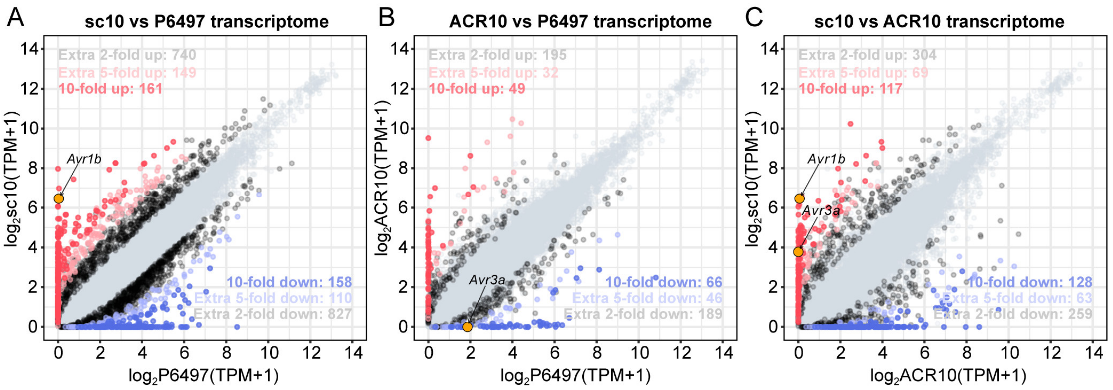

Figure S2. Comparative transcriptomic analysis of differential gene expression among sc10, P6497 and ACR10. Each dot represents the mean $\log_2$(TPM+1) value of a gene from biological replicates. Light grey dots indicate genes with no differential expression. Grey dots represent genes with a >2-fold change (|$\log_2$FoldChange| > 1, adjusted *P* value < 0.05). Light red and red dots indicate genes elevated by more than 5-fold and 10-fold, respectively, in the y-axis sample compared to the x-axis sample. Light blue and blue dots represent genes reduced by more than 5-fold and 10-fold, respectively. Differentially expressed *Avr* effector genes with a >10-fold change are highlighted as orange dots. Panels **A**, **B**, and **C** show comparisons between sc10 vs P6497, ACR10 vs P6497, and sc10 vs ACR10, respectively.

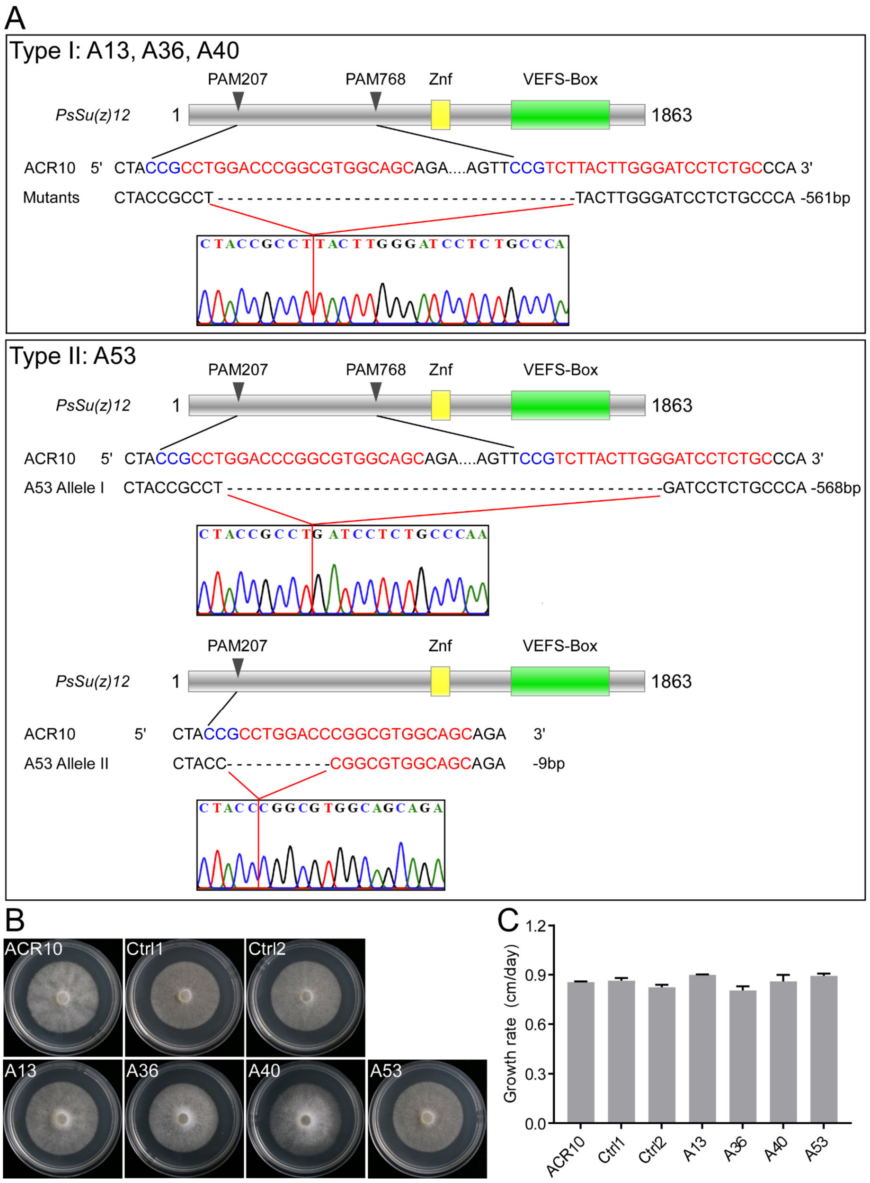

Figure S3. Generation and growth rate analysis of *pssu(z)12* mutants. (**A**) Schematic representation of *PsSu(z)12* mutations generated using CRISPR/Cas9 editing. PAM sites targeted by sgRNAs are indicated with black arrows. The flanking sequences at the editing sites and Sanger sequencing chromatograms confirming the mutations in the mutants are shown. (**B**) Vegetative growth of mutants shows no significant difference compared to ACR10. Mycelia were cultured on V8 medium plates for five days, and images were taken on the fifth day. (**C**) The diameters of mycelia were measured on the second and fifth days, and daily growth rates were calculated. Bars represent the mean fold changes (±SEM, n = 3). No significant differences were observed, as determined by Student's *t*-test.

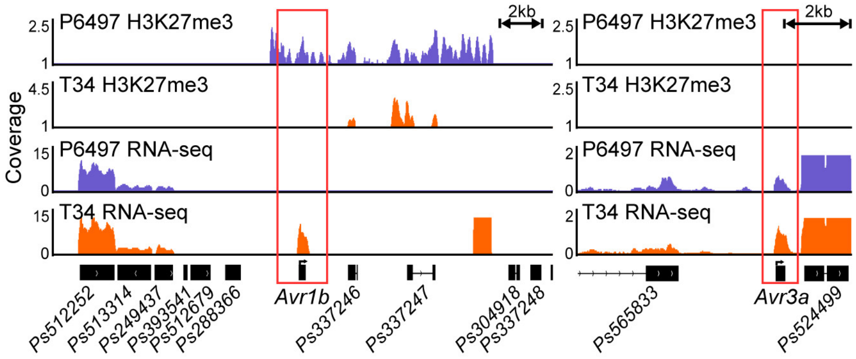

Figure S4. H3K27me3 ChIP-seq and RNA-seq data from the mycelia stage of P6497 and its *pssu(z)12* mutant T34 for the *Avr1b* and *Avr3a* loci are visualized in the IGV browser. H3K27me3 coverage was normalized to input as FE using MACS3, representing the mean value of biological replicates. The coverage of RNA-seq reads was normalized using BPM and merged as the mean value of biological replicates.

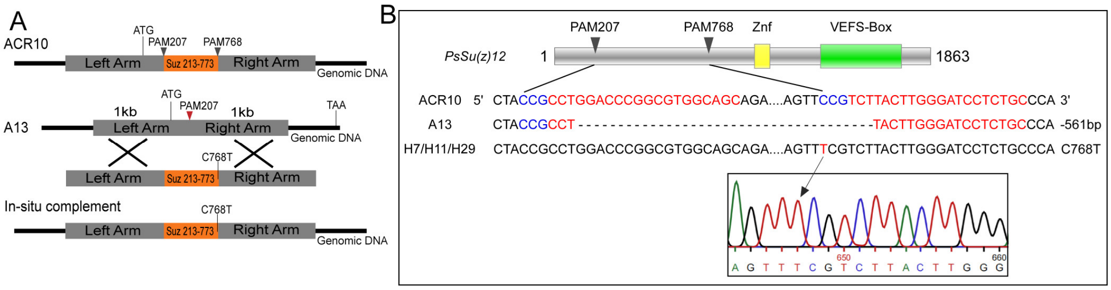

Figure S5. Schematic representation of *PsSu(z)12* complemented mutations generated using the gRNA-mediated HDR sequence replacement approach. (**A**) Diagram of the HDR strategy used for *PsSu(z)12* complementation. Homology arms flanking the target site were designed to replace the deleted region in A13 with the wild-type *PsSu(z)12* sequence containing a synonymous C768T mutation. The sgRNA207 used for ACR10 cutting and A13_sgRNA207 for A13 cutting share the same PAM207 site. (**B**) Sequence alignment showing the *PsSu(z)12* region in ACR10 (wild type), A13 (mutant), and complemented strains (H7, H11, H29). The PAM sites of gRNAs are highlighted in blue; the target regions are marked in red, and the synonymous C768T mutation is indicated. The sequencing trace of the complemented strains confirms the successful introduction of the mutation.

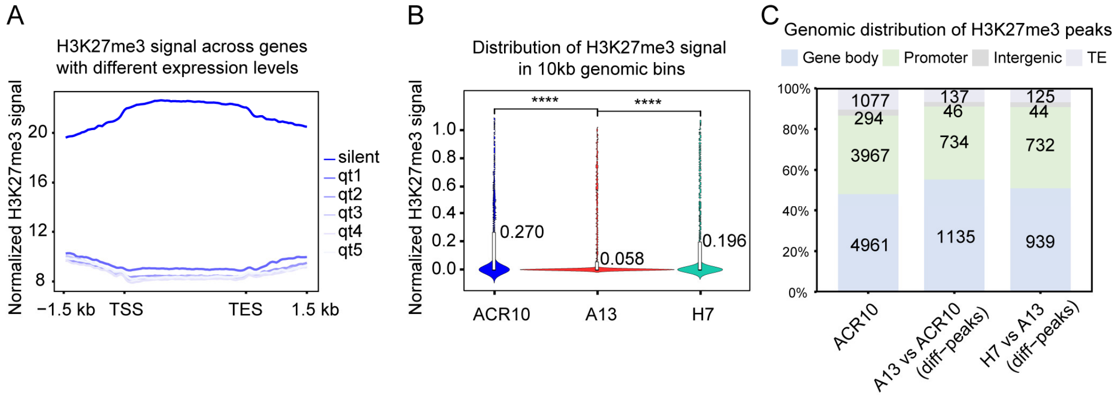

Figure S6. Genome-wide analysis of H3K27me3 distribution in *P. sojae*. (**A**) Normalized H3K27me3 signals across genes grouped by transcriptional levels. Genes with TPM < 0.1 were classified as transcriptionally silent ("silent"), while the remaining genes were divided into five quantiles (qt1–qt5) based on increasing expression. (**B**) A combination of violin plot and boxplot was used to illustrate global changes in H3K27me3 accumulation levels across the ACR10, A13, and H7 strains. H3K27me3 signals were normalized using Min-Max scaling to a range of 0 to 1 to enable direct comparison across strains. The third quartile (75th percentile) values are indicated. Statistical significance was assessed using a Kruskal–Wallis test for overall group differences, followed by pairwise Wilcoxon rank-sum tests. **** denotes $P < 0.0001$. (**C**) Genomic distribution of H3K27me3-enriched peaks across annotated features, including gene bodies, promoters, intergenic regions, and TEs. Numbers indicate the count of peaks in each category. "Diff-peaks" refer to differential peaks gained or lost in pairwise comparisons between strains.

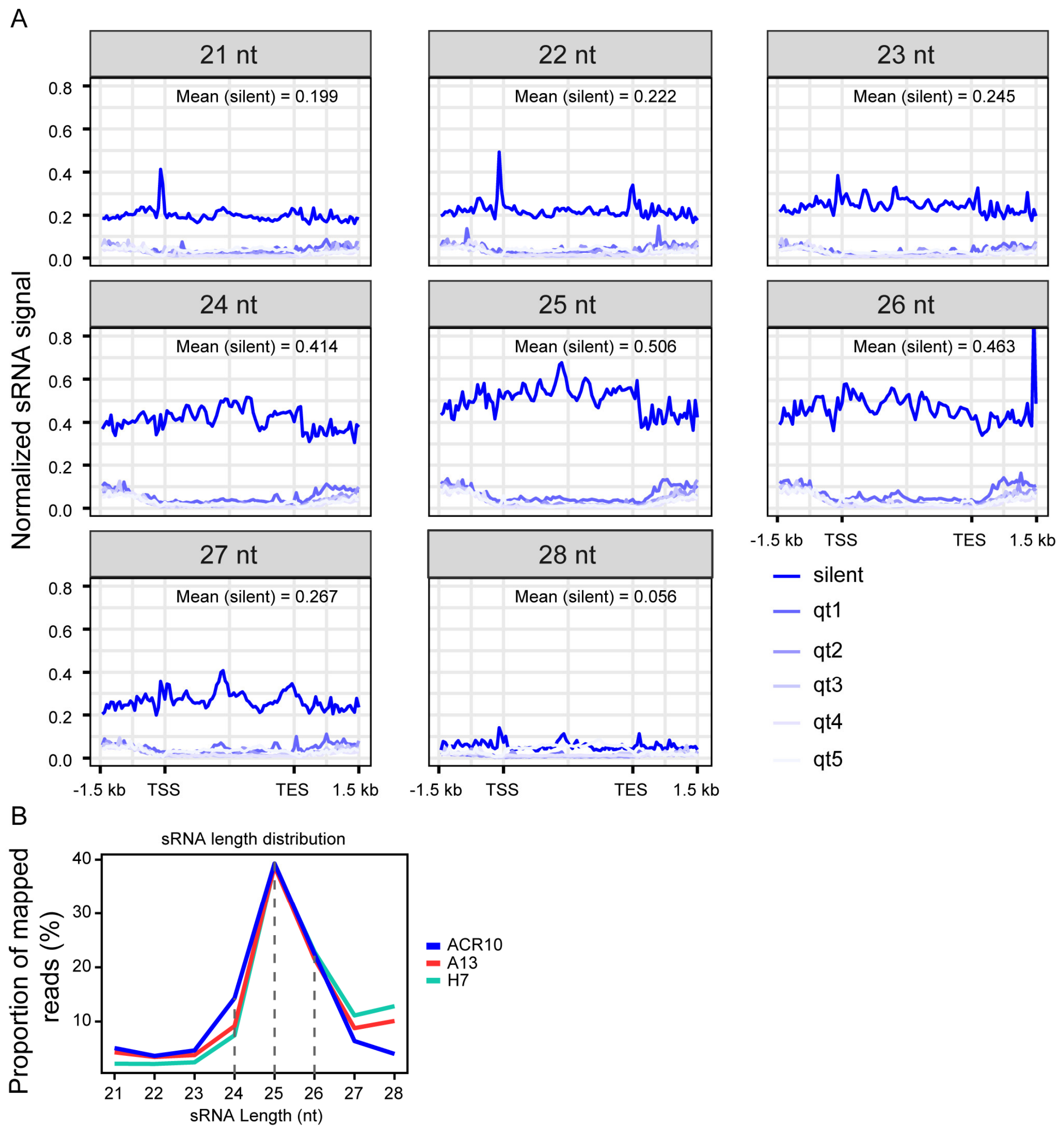

Figure S7. (**A**) Normalized sRNA signal (CPM) mapped across gene regions from -1.5 kb upstream of the TSS to +1.5 kb downstream of the TES, separated by sRNA length (21-28 nt). For each length category, the average signal for the "silent" group is indicated in panel. (**B**) Distribution of mapped sRNA reads by length across three strains.

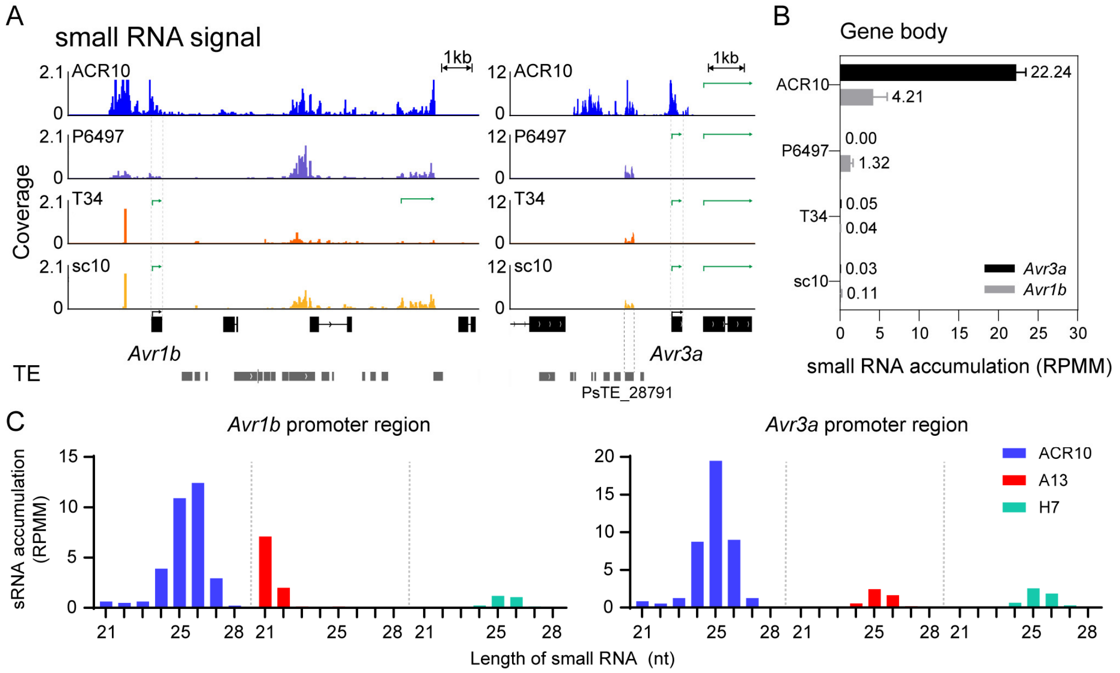

Figure S8. (**A**) sRNA-seq coverage (CPM) at the *Avr1b* and *Avr3a* loci in the P6497, T34 and sc10 strains. Green arrow indicates transcription. Predicted TE structures are shown. (**B**) sRNA accumulation levels (RPMM) over the gene body of *Avr1b* and *Avr3a* in different strains. (**C**) Length distribution of sRNAs mapping to the *Avr1b* and *Avr3a* promoter (1.5 kb) regions in ACR10, A13 and H7.

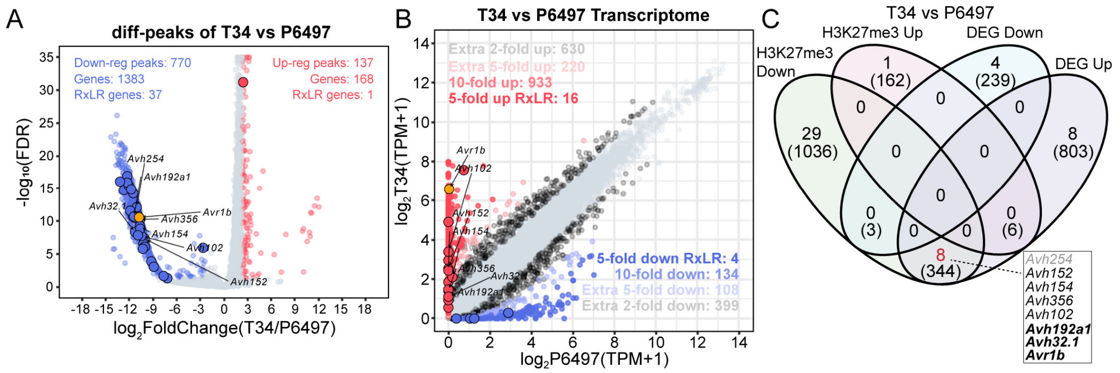

Figure S9. Integration of H3K27me3 and transcriptome data identifies RxLR effector genes regulated by H3K27me3 in P6497. (**A**) Volcano plots showing H3K27me3 five-fold differentially regulated peaks between T34 and P6497. Blue dots represent significantly downregulated peaks, while red dots represent significantly upregulated peaks. H3K27me3 differentially modified RxLR effector genes identified in panel (C) are highlighted. (**B**) Scatter plots of transcriptome comparisons between T34 and P6497. Gene expression is plotted as $\log_2$(TPM + 1). Red and blue dots represent genes with more than 10-fold upregulation or downregulation, respectively. Light red and light blue indicates extra 5-fold upregulated and downregulated genes, respectively. (**C**) The Venn diagram showing overlap between genes with five-fold H3K27me3 DMGs and five-fold DEGs in T34 vs P6497. The numbers indicate the count of genes in each category, with RxLR effector genes listed below the diagram. The gene *Avr1b* is

bolded, along with *Avh192a1* and *Avh32.1*, to denote overlap with RxLR effector genes identified in the ACR10 background. The gene *Avh254* is grayed out due to an erroneous gene model and was excluded from further analysis.

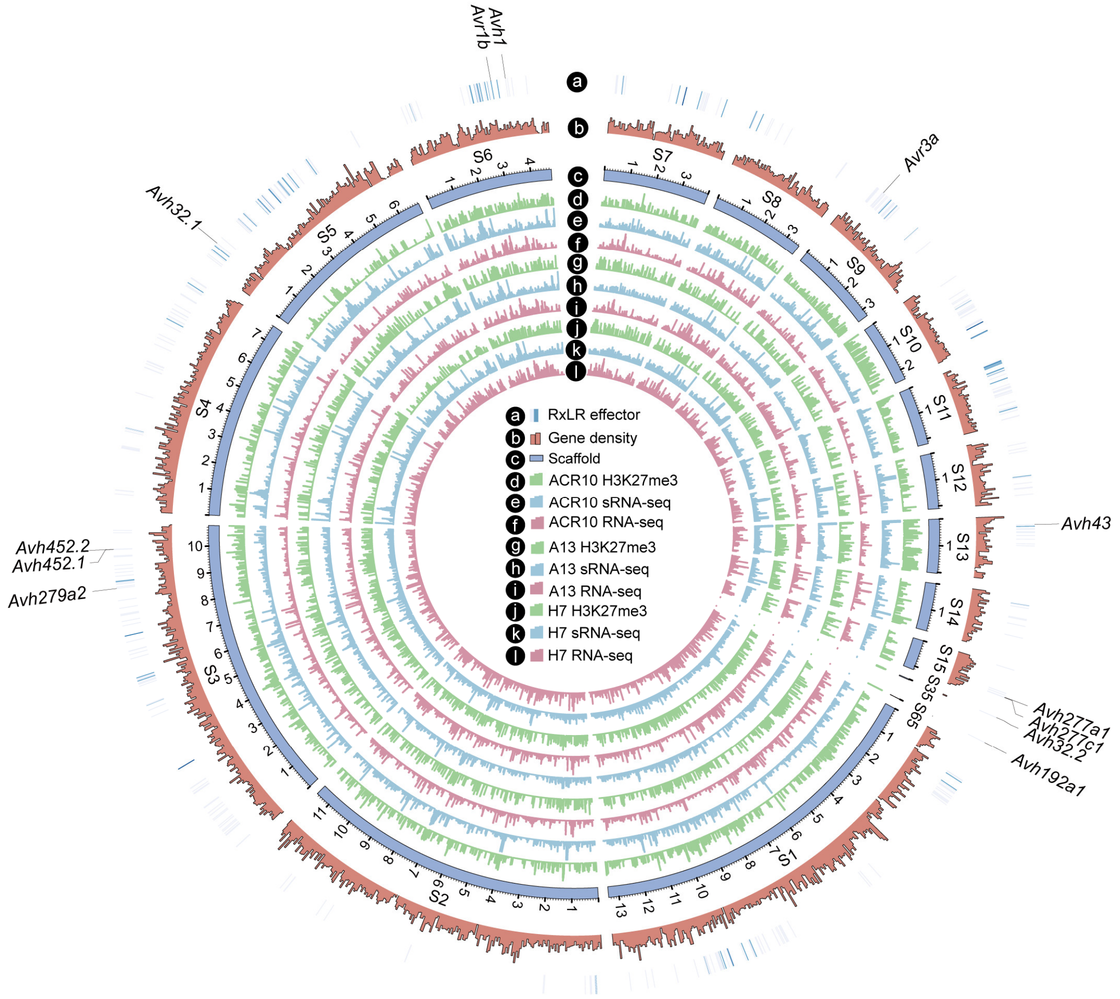

Figure S10. Circular genome map illustrating the distribution of H3K27me3 modifications and sRNAs in ACR10, A13 and H7. The *P. sojae* 3.0 genome was applied as reference genome in this study. **a** Blue lines represent the distribution density of RxLR effector genes in 10-kb windows. **b** gene density, total number of genes in 50-kb windows. **c** Scaffold tracks: the major ticks represent 500 kb, and the minor ticks represent 50 kb. **d, g, j** H3K27me3 chip-seq coverage were visualized based on FE value (normalized using base-2 logarithms) in 10-kb windows. **e, h, k** sRNA-seq coverage were visualized based on CPM values (normalized using the square root) in 10-kb windows. **f, i, l** RNA-seq coverage was visualized based on BPM values (normalized using base-2 logarithms, $\log_2(x + 1)$) in 10-kb windows.

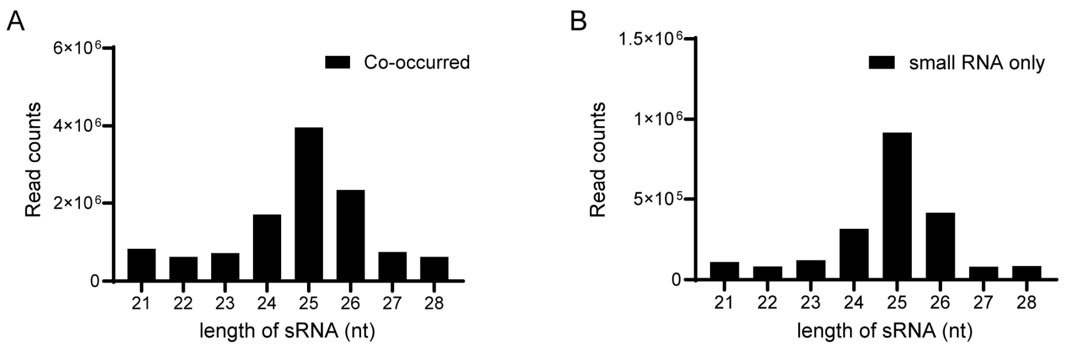

Figure S11. Length distribution of sRNAs mapping to genes of the co-occurred group (**A**) and the small RNA only group (**B**).

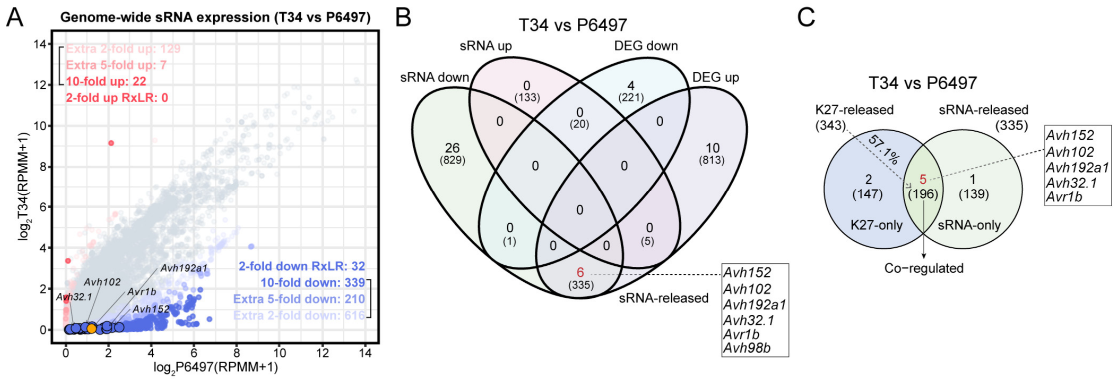

Figure S12. Analysis of differentially expressed sRNAs in ACR10. (**A**) Scatter plots of genome-wide sRNA expression in T34 vs P6497. Gene sRNA expression is plotted as $\log_2$(RPMM + 1). Blue and red dots represent genes with more than two-fold downregulation or upregulation, respectively. RxLR effector genes identified in panel (C) are noted. (**B**) Venn diagram showing overlap between genes with two-fold sRNA DEGs and five-fold DEGs in T34 vs P6497. 335 genes, including six RxLR effector genes, were identified as sRNA-released. (**C**) The Venn diagram illustrates the overlap between genes of K27-released and sRNA-released. Five-fold upregulated genes exhibiting downregulation of H3K27me3 without significant changes in sRNA levels were classified as K27-only, while genes showing downregulation of sRNAs without significant changes in H3K27me3 were classified as sRNA-only. A total of 196 overlapped genes (57.1% out of 343 K27-released) were classified as co-regulated. Five co-regulated RxLR effector genes are listed.

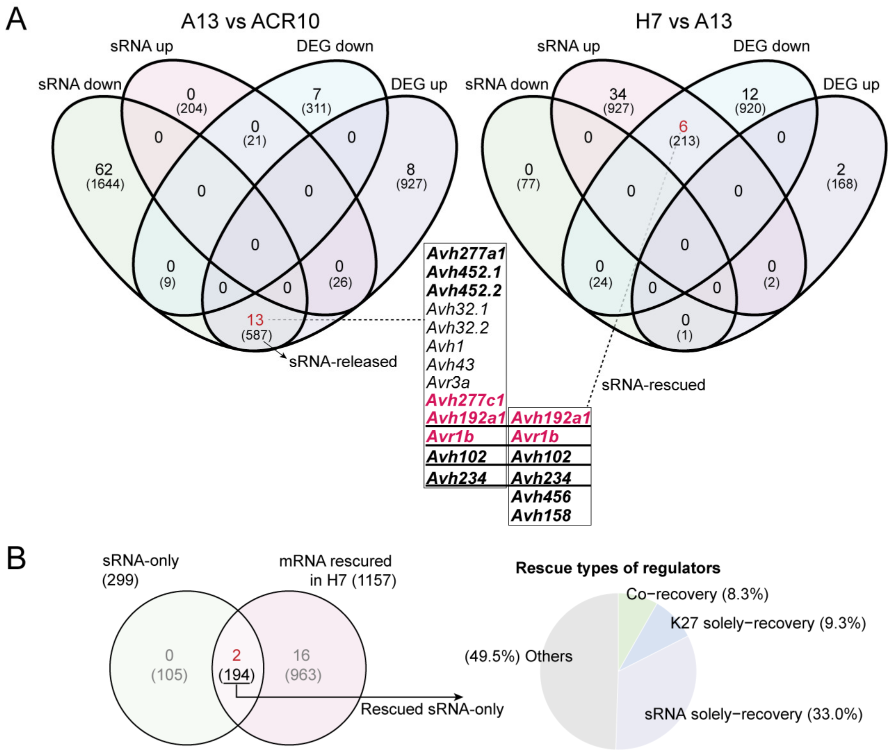

Figure S13. Differential analysis of sRNA expression in the ACR10 background. **(A)** Venn diagrams showing the overlap between genes with two-fold sRNA DEGs and five-fold DEGs in the comparisons of A13 vs ACR10 (left) and H7 vs A13 (right). The numbers indicate the corresponding RxLR effector genes, with the numbers in parentheses representing the total count of genes in each category. sRNA-released and sRNA-rescued RxLR effector genes are listed, with transcriptional differentially downregulated genes in H7 bolded. (**B**) The left Venn diagram illustrates the overlap between genes released by sRNA-only and those with mRNA rescue in H7. As a result, 194 genes were identified as rescued sRNA-only. The right panel shows the components of different recovery types of regulators.

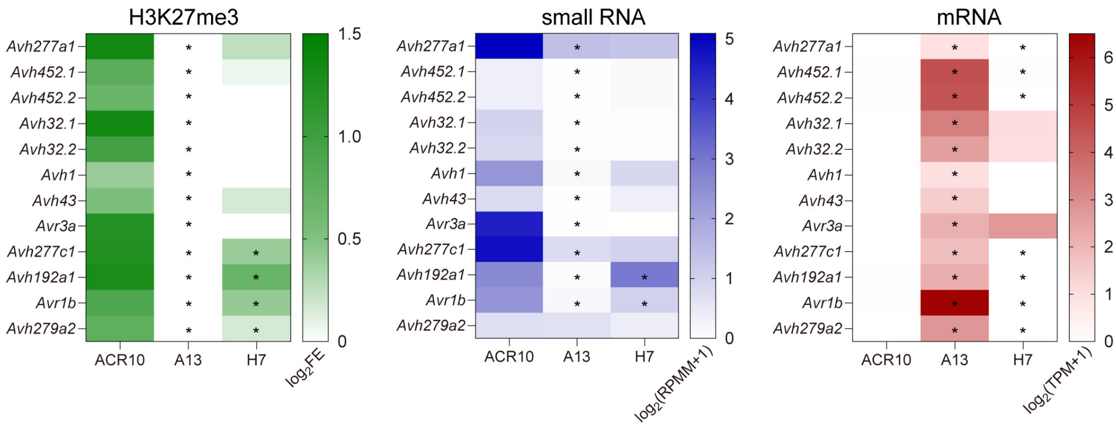

Figure S14. Heatmaps display fold enrichment of H3K27me3 (left, log_2FE), sRNA expression (middle, $log_2(RPMM + 1)$), and mRNA expression (right, $log_2(TPM + 1)$) for 12 RxLR effector genes highly correlated with H3K27me3 across ACR10, A13, and H7. Statistically significant genes are marked with an asterisk.

Supplementary Table S1. Primers used in this study.

Primer	Sequence	Function
AVR1B1F	CTGTACCATATCACCATCAGAG	Full length clone
AVR1B1R	GTGCCAATACCACCAAGTTGAG	
AVR3A1F	ATGCGCCTCGCTCAAGTTGTGG	
AVR3A1R	CTACGCGTTTTTCGCTGC	
AVR1BR1F	TCGAGCTCATGAAGAGGACG	RT-qPCR
AVR1BR1R	GGTCCGCGATTGCCAACCAG	
Sp92-33F (*Avr3a*)	GCTGCTTCCTTCCTGGTTGC	
Sp92-325R (*Avr3a*)	GCTGCTGCCTTTTGCTTCTC	
ACTAF	ACTGCACCTTCCAGACCATC	
ACTAR	CCACCACCTTGATCTTCATG	
PsSu(z)12-A13_sgRNA207F	CTAGCCTCTGCCTGATGAGTCCGTGAGGACGAAACGAGTAAGCTCGTCGCAGAGGATCCCAAGTAAGG	sgRNA construction
PsSu(z)12-A13_sgRNA207R	AAACCCTTACTTGGGATCCTCTGCGACGAGCTTACTCGTTTCGTCCTCACGGACTCATCAGGCAGAGG	
HDR_suzup794F	ACTAGTGGATCCCCCCTCTTGGGCAGTCGTACAGG	HDR donar plasmid construction
SuzC768T_R	GAGGATCCCAAGTAAGACGAAACTCGCACTTGACGCCA	
SuzC768T_F	TCGTCTTACTTGGGATCCTC	
Suz1771-HDR-R	GAATTCCTGCAGCCCCACTTCTTCATGAACTCCTT	
Suz94F	GAGCACATGGAGGAGGAGTTC	Transformants screening
Suz1817R	ATTCGCGTTGCTGATACCCA	
NC dsRNA (sense)	CAACACAUAACAGGACAGAUACGAG	dsRNA synthesis
NC dsRNA (anti-sense)	CUCGUAUCUGUCCUGUUAUGUGUUG	
Avr3a Si79 (sense)	AACCAGGCCAAGAUCAUCAAAGGAA	
Avr3a Si79 (anti-sense)	UUCCUUUGAUGAUCUUGGCCUGGUU	
Avr3a Si83 (sense)	AGGCCAAGAUCAUCAAAGGAA	
Avr3a Si83 (anti-sense)	UUCCUUUGAUGAUCUUGGCCU	

Supplementary Table S2. Classification of silencing modes and mechanisms.

Gene category	Observed silencing mode	Proposed silencing mechanism	Genes identified upon *PsSu(z)12* editing	
			RxLR effector	Whole genome
**K27-only**	On-off switch	TGS	1	197
**sRNA-only**	Dose-dependent	PTGS	2	299
**Co-occurred**	On-off switch	TGS / TGS - PTGS	11	288
